# Collagen secretion and maturation in osteogenesis imperfecta: Systematic review and meta-analysis

**DOI:** 10.1016/j.bonr.2026.101928

**Published:** 2026-06-08

**Authors:** Priyesh Patel, Sirion Aksornthong, Svetlana V. Komarova

**Affiliations:** a Faculty of Dental Medicine and Oral Health Sciences, McGill University, Montréal, Canada; b Shriners Hospital for Children – Canada, Montréal, Canada; c Department of Experimental Surgery, McGill University, Montreal, QC, Canada; d Department of Biomedical Engineering, Faculty of Engineering, University of Alberta, Edmonton, Alberta, Canada

**Keywords:** Osteogenesis imperfecta, Type 1 collagen, Secretion, Fibril diameter, Crosslink, Rare disease, meta-analysis

## Abstract

Osteogenesis imperfecta (OI) is a rare genetic disorder most often caused by mutation in genes that encode collagen type I. The collagen pathway is a complex process involving collagen folding, secretion and fibril assembly. The underlying mutation of OI causes a cascade effect resulting alterations at all levels of the collagen pathway. The objective of this study is to use knowledge synthesis to quantify the collagen-I folding kinetics, secretion kinetics, crosslinks, fibril diameter and melting temperature. A systematic search in Medline, Ovid and Web of Science, identified 1001 studies reporting on selected outcomes in OI patients. After screening, we included 51 qualitative studies, 8 quantitative and 43 studies for meta-analysis. Meta-analysis of studies with quantitative data was performed using normalized mean difference as a study-level effect size and a random-effects model with the Hunter and Smith with sample size correction. The collagen-I folding half-life dataset included 8 patients across 3 studies and had an effect size of 0.88 (confidence interval (CI) 0.31, 1.46). The collagen secretion half-life dataset included 8 patients across 7 studies and had an effect size of 0.23 (CI: −0.13, 0.59). The collagen crosslink dataset included 44 patients across 2 studies and had an effect size of 0.37 (CI: 0.09, 0.65). The fibril diameter dataset included 168 patients across 11 studies and had an effect size −0.13 (CI: −0.24, 0.02). The melting temperature dataset is expressed as absolute mean difference, it included 85 patients across 26 studies and had an effect size of −2.29 (CI: −3.32, −1.35). These findings show the collagen pathway is altered in OI, beyond the initial mutation. Our study provides new insights into collagen-I pathophysiology in OI, generating new hypotheses regarding the collagen pathway and mediating disease presentation in different tissues and overall severity.

## Highlights

• Mutations and biochemical changes alter collagen-I in osteogenesis imperfecta
• We analysed collagen-I secretion and maturation in 329 OI patients from 43 studies
• Collagen-I folding and secretion occurred slower in OI
• OI collagen-I demonstrated higher crosslinking but smaller fibril diameter
• Altered collagen-I processing and fiber assembly may contribute to OI severity

## Graphical abstract

**Figure:**
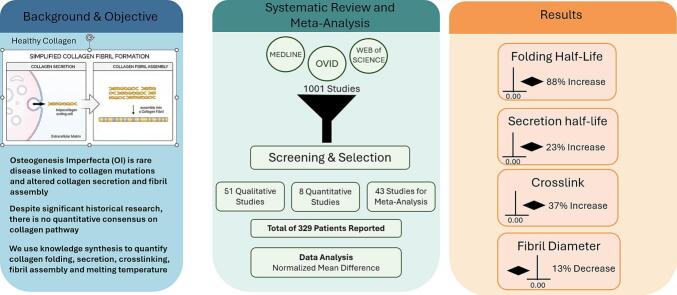

## 1 Introduction

Osteogenesis imperfecta (OI) is a rare metabolic bone disease, occurring in 1 in 15,000 births. The majority of cases (∼85%) result from mutations in one of the two genes that encode type I collagen, COL1A1 and COL1A2 (Rauch and Glorieux, 2004; Tournis and Dede, 2018; Nijhuis et al., 2019; Tauer et al., 2019). Being the most prevalent protein in bone tissue, the most significant clinical manifestation of OI is bone fragility. Additionally, OI has other musculoskeletal manifestations, including skeletal deformities, scoliosis, low bone mineral density, and dentinogenesis imperfecta (Tournis and Dede, 2018). OI caused by collagen-I mutations is traditionally classified into four clinical types (I–IV): type I is mild; type II is the most severe and is often perinatal or neonatal lethal; type III is severe; and type IV is considered moderate-to-severe (Van Dijk et al., 2010).

Collagen-I is a heterotrimer composed of two COL1A1 chains and one COL1A2 chain assembled into a triple helix (Buss et al., 2022). The collagen-I pathway is a complex multistep process spanning intracellular biosynthesis, secretion, extracellular processing, and matrix assembly (Revell et al., 2021). Following translation in the endoplasmic reticulum, procollagen chains undergo post-translational modification and chaperone-mediated triple-helix folding (Makareeva et al., 2011). Properly folded procollagen is then trafficked through the Golgi and secreted via vesicular export (Malhotra and Erlmann, 2015). In the extracellular space, procollagen undergoes N- and C-propeptide cleavage to generate tropocollagen, which can self-assemble into fibrils. Fibrillogenesis is further shaped by extracellular matrix interactions and enzymatic cross-linking, particularly through lysyl oxidase-mediated reactions, which regulate fibril maturation, fibril diameter, and tissue mechanical properties (Garnero, 2015). Collagen stability can also be assessed by melting temperature, a measure of triple-helical integrity (Nijhuis et al., 2019). In OI, pathogenic collagen mutations have been reported to alter several stages of this pathway, including secretion, cross-linking, fibril diameter, and melting temperature. Over the years many case studies have characterize these outcomes. However, quantitative consensus on the magnitude of these alterations remains limited, and their relationship to disease severity is not yet fully defined.

We performed a systematic review and meta-analysis on OI collagen-I secretion and maturation. The primary objective was to provide descriptive quantitative synthesis of data describing folding, secretion, crosslinks, fibril diameter and melting temperature levels in OI collagen-I compared to healthy controls. The secondary objective was to examine the association between selected outcomes and disease severity.

## 2 Materials and methods

### 2.1 Information sources, search strategy, eligibility criteria, and screening

This study complies with the Preferred Reporting Items for Systematic Review and Meta-Analysis (PRISMA) statement (**Supplementary Table 1**) (**Page et al., 2021****)**. The search strategy used combined keywords and Medical Subject Headings (MESH) term related to OI and collagen terms related to selected outcomes (folding, secretion, crosslinks, fibril diameter and melting temperature). The search was performed in Medline, Embase, and Web of Science in January 2023 and updated in January 2025. The systematic review was not registered in PROSPERO. The title and abstract screening was performed independently by two reviewers (PP and SA) using the Rayyan Systematic Review Screening Software and disagreements between screeners were resolved by discussion (Ouzzani et al., 2016). The full text screening was performed by PP with the inclusion criteria of studies assessing the selected outcomes for OI collagen. The included studies were grouped as i) studies containing a qualitative assessment of selected outcomes of OI collagen; ii) studies reporting quantitative data excluded for meta-analysis on selected outcomes of OI collagen; iii) studies reporting quantitative data included for meta-analysis on selected outcomes of OI collagen. The exclusion criteria were studies describing OI types that are not due to collagen-I mutations, animal studies, conference abstracts, reviews and editorial commentary. The full list of included studies is in the **Supplementary bibliography**.

### 2.2 Data extraction

Publication information and patient parameters were extracted from studies in all three groups: meta-analysis, quantitative and qualitative. It includes authors, publication year, number of patients/controls, patient OI type (collagen-I mutation when reported), age and sex of participant. Some studies did not explicitly state the patient's OI type; however, the disease severity could be determined from the patient's description (e.g., perinatal lethal OI type II). Secretion and melting temperature are outcomes that present qualitative data. For both outcomes, The Ymax and melting temperature were noted as lower, equal or higher than the control. For quantitative secretion, the Ymax of the curve is annotated as the Ymax lower, equal or higher than control. For quantitative folding data, the total folding time was annotated from the graph as lower, equal or higher than control. Each outcome for meta-analysis has its own study-reported units. For collagen folding, the half-life and exponential factors parameters were extracted from the curve using OriginPro. Collagen-I secretion half-life, exponential factor and Ymax parameter were extracted from the curve using OriginPro. **Supplementary Fig. S1** describes the process of extracting outcome parameters from the software. For collagen-I crosslink outcome, the hydroxylysylpyridinodine (HP) and lysylpyridinodine (LP) was reported as “total” crosslink in the form of HP + LP. For Studies reporting individual crosslink data in figures, a plot digitizer was used to extract the quantitative data. For collagen fibril diameter, the diameter measurement in nanometers was noted, and the origin of the sample (osteoid and dermis) was noted. The melting temperature was recorded in degrees Celsius, and the instrument used to measure it was specified.

### 2.3 Study level outcomes

All publications presented individual patient data (except when mentioned otherwise). The selected effect size for all outcomes except for melting temperature was the normalized mean difference, calculated as $\theta_{i}=\frac{\theta_{i}^{p}-\theta_{i}^{c}}{\theta_{i}^{c}}$, where $\theta_{i}^{p},\;\theta_{i}^{c}$ were means for the patient and control populations in study *i (*Mikolajewicz and Komarova, 2019*).* For fibril diameter outcome, some publications presented data as the means per OI type, in which case a weighted average was calculated for $\theta_{i}^{p}$. For the melting temperature, the selected effect size was the absolute mean difference $\theta_{i}=\theta_{i}^{p}-\theta_{i}^{c}$, where $\theta_{i}^{p},\;\theta_{i}^{c}$ were means for the patient and control populations in study *i.*

To estimate the standard errors, we used the following considerations. For studies that reported individual patient data (IPD) for 4 or more patients or controls per outcome (2 of 2 studies for crosslink dataset and 1 of 26 studies for melting temperature) the standard deviations for patients and controls were calculated as $\;\mathrm{sd}\left(\theta_{i}\right)=\sqrt{\frac{\sum_{i=1}^{n}{\left(x_{i}-\bar{x}\right)}^{2}}{n-1}}$, where $x_{i}$ represents individual datapoints, and $\bar{x}$ is the mean of individual datapoints. To estimate the SD for samples in studies with fewer than 4 patients or controls (3 of 3 studies for folding dataset, 7 of 7 studies for secretion and 6 of 11 studies for fibril diameter), first, we calculate the combined SD for all data points in small-sized studies, treating data for patients and controls separately. Then, the combined SD for patients was imputed to patients or patient groups in each study with 4 or fewer patients, while the combined standard deviation for controls was assigned to controls or control groups in each study with 4 or fewer controls (Furukawa et al., 2006; Seo et al., 2021). For the 5 of 11 studies in the fibril diameter dataset the absolute mean difference is used as the OI type, the between group variance was used $\mathrm{sd}\left(\theta_{i}\right)=\frac{\sum_{j}n_{j}{\left({\bar{x}}_{j}-\bar{x}\right)}^{2}}{N-1}$, where $n_{j}$ is the sample size of group, ${\bar{x}}_{j}$ is the mean of the group, $\bar{x}$ is the overall mean across groups, N is the total sample size across all groups. To calculated the study-level standard error was calculated as $\mathrm{se}\left(\theta_{i}\right)=\sqrt{\frac{{\left(\frac{\mathrm{sd}\left(\theta_{i}^{c}\right)}{\theta_{i}^{c}}\right)}^{2}}{n_{i}^{c}}+\frac{{\left(\frac{\mathrm{sd}\left(\theta_{i}^{p}\right)}{\theta_{i}^{p}}\right)}^{2}}{n_{i}^{p}}\;}$, where $\mathrm{sd}\left(\theta_{i}^{c}\right)\;\mathrm{and}\;\mathrm{sd}\left(\theta_{i}^{p}\right)\;$ are the SD from the pooled control and patient data, $n_{i}^{c}\mathrm{and}\;n_{i}^{p}$ is the number controls and patients within the study. The melting temperature dataset uses a different formula for standard error because absolute mean difference is used as effect size. The same approach as the other dataset with 4 or less patients was used for the melting temperature dataset (25 of 26 studies for melting temperature). The standard error is calculated using the following formula $\mathrm{se}\left(\theta_{i}\right)=\sqrt{\frac{\mathrm{sd}{\left(\theta_{i}^{c}\right)}^{2}}{n_{i}^{c}}+\frac{\mathrm{sd}{\left(\theta_{i}^{p}\right)}^{2}}{n_{i}^{p}}}$, where $\mathrm{sd}\left(\theta_{i}^{c}\right)\;\mathrm{and}\;\mathrm{sd}\left(\theta_{i}^{p}\right)\;$ are the SD from the pooled control and patient data, $n_{i}^{c}\mathrm{and}\;n_{i}^{p}$ is the number controls and patients within the study.

### 2.4 Meta-analysis

Meta-analysis was performed using a random effects model. The global effect size ($\hat{\theta })$ and its standard error was calculated using the Hunter-Schmidt estimator with small sample size correction (Riley et al., 2011). Confidence intervals (CI) were calculated as $95\%\mathrm{CI}=\hat{\theta }\pm Z_{\left(1-\frac{\alpha }{2}\right)}\times \mathrm{SE}\left(\hat{\theta }\right)=\hat{\theta }\pm 1.96\times \mathrm{SE}\left(\hat{\theta }\right)$. Heterogeneity analysis was performed, and the percentage of variation across studies due to heterogeneity, I^2^, and between-study variance, τau^2^, were reported.

To evaluate the covariate effect, the individual effect size was calculated by comparing the individual patient value with the average control value. Then, the individual patient effect sizes were combined by their covariates (Lambert et al., 2002). Only studies in which the analyzed covariates were explicitly reported were included in this analysis.

### 2.5 Assessment of bias

Studies selected for meta-analysis were evaluated for bias using a quality assessment tool that includes questions to assess case studies. The assessment consists of 10 questions, with a maximum obtainable score of 11 points (**Supplementary Methods 2**). A funnel plot was made for each outcome (**Supplementary Figure S2**) to visualize study-level reporting bias (Viechtbauer and Cheung, 2010).

### 2.6 Software

EndNote 20 and Rayyan were used for reference management (Ouzzani et al., 2016). Webplot digitizer was used for data extraction from figures (melting temperature DF). OriginPro 2025b was used for curve fitting folding and secretion curves to determine Ymax, half-life and exponential factor using hill fit. Microsoft Excel version 2507 build 16.0.19029 was used for data management, effect size and standard error calculations. Python with the matplotlib package was used to make some figures (Hunter, 2007). R version 4.2.2 was used in Visual Studio Code version 1.103.1 with the metafor package for meta-analysis (forest plot, heterogeneity analysis, funnel plot), and the tidyplots package for figure preparation (Engler, 2025; Viechtbauer, 2010).

## 3 Results

**Fig. 1:**
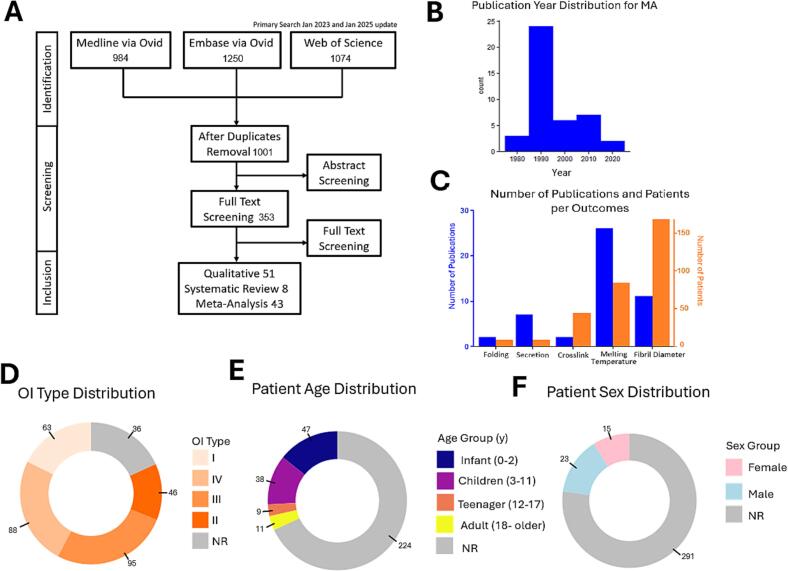
**Systematic review and meta-analysis information flow.** (A) The PRISMA diagram indicates the flow of assessment in each step of the process. (B) The publication year distribution for all included studies. (C) Bar plots for number of publications (blue, left y-axis) and patients (orange, right y-axis) for meta-analysis of collagen secretion and maturation for folding, secretion (SEC), crosslink (CRO), melting temperature (MT) and fibril diameter (FD). (D, E, F) Pie charts visualizing reporting of patient parameters including OI type (D), age (E), and sex (F). NR: not reported.

A systematic search was conducted across three databases: Medline (*n* = 984), Embase (*n* = 1250), and Web of Science (*n* = 1074), which after duplicate removal, identified 1001 unique studies. These studies were screened by title and abstract, and 353 were selected for full-text evaluation (Fig. 1A). After meeting the inclusion criteria of describing desired outcomes for collagen-I in OI patients, 102 studies primarily published in the early to late 90s (Fig. 1B) were included in the systematic review. Of these, 51 studies provided only qualitative data, 8 reported quantitative outcomes that could not be combined with other studies, and 43 were included in the meta-analysis (complete reference lists are in the **Supplemental bibliography**). The meta-analysis comprised studies assessing outcomes related to collagen-I secretion and maturation in OI, including 3 studies with 8 patients for collagen-I folding, 7 studies with 8 patients for collagen secretion, 2 studies with 44 patients for collagen crosslink, 11 studies with 168 patients for fibril diameter and 26 studies with 85 patients for collagen melting temperature (Fig. 1C). A total of 329 patients was described in meta-analysis, 70 patients in qualitative analysis and 16 patients in quantitative analysis. The OI type distribution was balanced across all four types, and for 36 patients, the OI type was not reported (NR) (Fig. 1D). The age was not reported for the majority of patients; when reported, it was mainly for the pediatric population (Fig. 1E). The patient sex was only reported for 38 of 329 patients (Fig. 1F).

Of the 43 quantitative studies included in the meta-analysis, 24 were case reports with 1 patient each, 11 were case series with 2–4 patients per study, and 3 were cohort studies with 12–62 patients per study (Table 1). The number of control samples was often limited to a single sample, except in certain cohort studies. The study-level effect sizes were calculated as normalized mean differences for all outcomes except melting temperature, for which we used absolute mean differences. Values higher than zero indicate a higher level in OI patients, and values lower than zero indicate a lower level in OI patients. For studies reporting aggregate data or IPD for more than 4 patients and controls, we used the reported or calculated mean and SD to estimate the study level effect size and its variance. For studies reporting 1–3 patient or control samples, we calculated the global variance for both patients and controls by combining the SD from all the studies reporting the same outcome. The global variance was then imputed to the patients and controls in relevant studies, followed by calculation of the effect size and its variance (standard error). Next, the specific parameters of collagen-I secretion and maturation were analyzed.

**Table 1:** **Reported outcomes and quality assessment of studies included in meta-analysis**. Shown are the publications and their respective outcomes, and quality scores (QS; out of 11 maximum).

Author and Year	Outcome	Quality Score
Kirsch, 1981	Melting Temperature	6
Steinmann, 1984	Diameter, Melting Temperature	6.5
De Vries, 1986	Secretion, Melting Temperature	5.5
Vogel, 1987	Secretion, Melting Temperature	7
Constantinou, 1987	Melting Temperature	4.5
Tenni, 1988	Melting Temperature	7.5
Rao, 1989	Melting Temperature	8
Baker, 1989	Melting Temperature	7
Baker, 1989	Melting Temperature	7
Constantinos, 1989	Melting Temperature	7
Baldwin, 1989	Melting Temperature	7.5
Valli, 1990	Melting Temperature	8
Valli, 1991	Secretion, Melting Temperature	7
Bateman, 1991	Melting Temperature	5
Wenstrup, 1991	Melting Temperature	7
Bonaventure, 1992	Secretion	7
Bateman, 1992	Melting Temperature	7
Bateman, 1992	Melting Temperature	8
Edwards, 1992	Melting Temperature	8
Mottes, 1993	Secretion	3.5
Brenner, 1993	Diameter	8
Valli, 1993	Secretion	7.5
Vetter, 1993	Crosslink	8
Kurosaka, 1994	Melting Temperature	8
Raghunath, 1994	Folding	8
Sarafova, 1998	Melting Temperature	6.5
Bank, 2000	Crosslink	7
Cabral, 2001	Melting Temperature	4
Cabral, 2003	Melting Temperature	6
Cabral, 2007	Melting Temperature	7
Kruczek, 2008	Melting Temperature	7
Makareeva, 2008	Melting Temperature	4.5
Taga, 2013	Melting Temperature	4
Mirigian, 2014	Folding	4.5
Mirigian, 2014	Secretion	4.5
Makareeva, 2018	Folding	4
Makareeva, 2018	Melting Temperature	6
Sarathchandra, 1999	Diameter	5
Vomund, 2004	Diameter	4.5
Balasubramanian, 2015	Diameter	3.5
Jones, 1984	Diameter	3
Stoss, 1993	Diameter	5.5
Barnes, 2019	Diameter	4.5
Cassella, 1991	Diameter	6
Cassella, 1994	Diameter	6.5
Lindahl, 2011	Diameter	5.5

### 3.1 Collagen folding

The pulse-chase experiment uses a metabolic label ([^35^S]methionine or azidohomoalanine (AHA)) to expose cells to the label, then removes it to track folding (Mirigian et al., 2014; Raghunath et al., 1994). The cell is lysed and passed through SDS-page to quantify the signal by densitometry. Raghunath et al. used [^35^S]methionine label (Raghunath et al., 1994). There was no difference in expected outcomes among the methods, and both were performed at 37 °C using fibroblasts. The data is presented as a percentage folded over time graph. The change in collagen in folding outcome measured the folding half-life and the exponential factor.

Quantitative studies for folding comprise 1 study with 3 patients Table 2. The assay was not carried to completion, as some patients do not start at 0% folded and end at 100% folded. All three patients seem to have a lower folding rate (slope of the curve) than the control.

**Table 2:** **Overview of studies containing quantitative data excluded from meta-analysis for folding outcomes.** Total of 3 patients.

Author and Year	Shape of curve	Decrease in Ymax	Reason for exclusion from meta-analysis
Barnes, 2019	Linear	Yes	Linear curve appearance as oppose to the expected sigmoidal curve. Assay doesn't start at 0 for control and assay doesn't reach 100% folded state for patients.

**Fig. 2:**
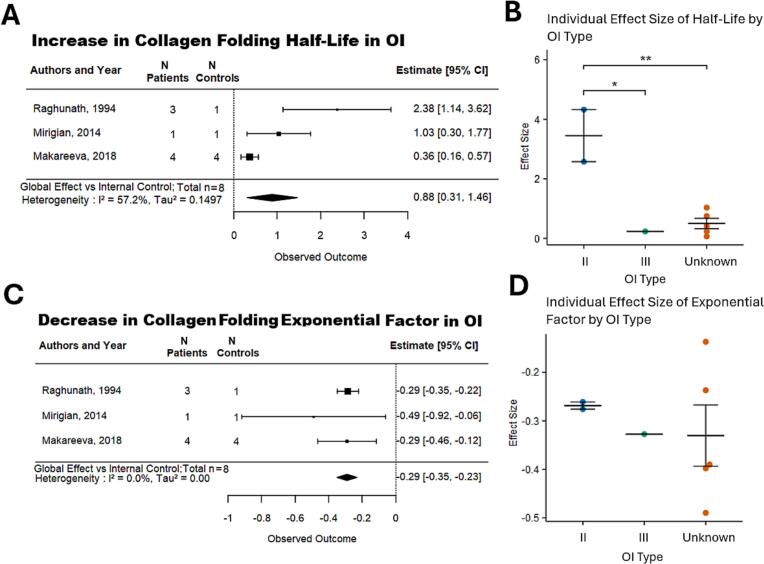
**Collagen folding of OI patients compared to controls.** (A, B) Forest plots of percentage difference in OI collagen-I folding half-life (A) and exponential factor (B) compared to control; positive values indicate higher levels in patients. Squares with lines depict the study-level effect, normalized mean difference with 95 % Cl; the size of the square is proportional to the study weight. The diamond represents the global effect size. Heterogeneity statistics I^2^ and τau^2^ are reported. (C, D) Collagen-I folding half-life (C) and exponential factor (D) in individual patient data separated by OI type, Unknown are samples in which OI type is unclear. Shown are individual data effect size points and their mean ± SEM; *p < 0.05 and ** *p* < 0.01 indicate significant differences by 1-way ANOVA with Tukey's post hoc test.

The meta-analysis included 3 studies describing 8 patients and demonstrated a significant overall increase in half-life, with a global effect size of 0.88 [95% CI: 0.31, 1.46] (Fig. 2A). Heterogeneity was minimal, with τau^2^ estimated at 0.15 and I^2^ at 57%. Individual data subgroup analysis suggested that OI type II (the most severe) may have a higher half-life compared to type III and unknown OI type (Fig. 2B). The meta-analysis on collagen folding exponential factor demonstrated an overall decrease compared to healthy controls, with a global effect size of −0.29[95% CI: −0.35, −0.23] (Fig. 2C**)**. The individual data subgroup analysis by OI type didn't show correlation with OI severity (Fig. 2D**)**. It is important to state that the individual subgroup analysis by OI analysis is severely underpowered by the lack of data as only 3 out of the 8 patients have their OI type reported in this outcome. The Raghunath et al. study included in the meta-analysis sampled 3 OI patients with glycine to cysteine mutation at different positions and suggested that mutations closer to the C terminus are associated with slower overall folding (Raghunath et al., 1994). Overall, the half-life analysis and exponential factor analysis indicate that OI collagen folds more slowly than the control.

### 3.2 Collagen secretion

The pulse experiment uses [^3^H] proline as a metabolic label to measure the collagen secretion. The cell medium is passed through SDS-PAGE to quantify the signal by densitometry (Valli et al., 1993). The data is presented as a percentage secreted over time. The change in collagen-I secretion outcome measured the secretion half-life, the exponential factor and Ymax.

The qualitative dataset for collagen secretion comprises 21 studies with 40 patients (Table 3). These studies have reported slower collagen secretion by collecting collagen from the culture medium of fibroblast cultures. Using gel electrophoresis, studies have compared the opacity of the OI band to a control to determine if there is less collagen in the media (Byers et al., 1988). This is reported in the Table 1 as lower Ymax. All studies have reported a lower Ymax.

**Table 3:** **Overview of studies containing qualitative data for secretion outcome**. Shown are the number of patients (NP) and controls (NC), OI type or reported severity, patient's age (in years, y) and sex (male (M), female (F), not reported (NR)), method, Ymax refers to lower total secretion amount. Total of 29 patients.

Author and Year	NP	NC	OI Type	Age (y)	Sex	Method	Ymax
Barsh, 1982	2	1	1	24 &28	M, F	Electrophoresis	lower
Wenstrup, 1986	1	1	3	8	M	Electrophoresis	lower
Byers, 1988	1	1	2	infant	NR	Electrophoresis	lower
Willing, 1988	1	1	2	infant	NR	Electrophoresis	lower
Bateman, 1989	1	1	2	infant	NR	Electrophoresis	lower
Marini, 1989	1	1	4	4	F	Electrophoresis	lower
Wallis, 1990	1	1	2	infant	NR	Electrophoresis	lower
Bateman, 1991	1	1	4	NR	NR	Electrophoresis	normal
Wallis, 1992	1	1	2	infant	M	Electrophoresis	lower
Bateman, 1992	1	1	2	infant	NR	Electrophoresis	lower
Superti-Furga, 1993	1	1	1	5	M	Electrophoresis	lower
Valli, 1993	1	1	4	8	M	Electrophoresis	lower
Constantinou-Deltas, 1993	1	1	2	infant	NR	Electrophoresis	lower
Mundlos, 1996	2	1	3,4	11,13	M, F	Electrophoresis	lower
Nicholls, 1996	1	1	NR	8	NR	Electrophoresis	normal
Oliver, 1996	1	1	3	3	F	Electrophoresis	lower
Pace, 2001	1	1	2,3,4	NR	NR	Electrophoresis	lower
Pace, 2001	1	1	1	6	M	Electrophoresis	lower
Augusciak-Duma, 2018	3	1	NR	NR	NR	Electrophoresis	normal
Lindert, 2018	1	1	2	infant	NR	Electrophoresis	lower
Takeyari, 2021	6	1	1,3	NR	NR	Elisa	lower

Quantitative secretion studies comprise 7 studies and 12 patients. A detailed breakdown of why each publication wasn't included in the meta-analysis is described in Table 4. All patients except three from the Besio et al. paper have a lower Ymax than the control (Besio et al., 2018a). Some studies suggested (de Vries and de Wet, 1986; Wallis et al., 1990) that in some heterozygous COL1A1 mutations, collagen-I trimers containing different numbers of mutant chains may differ in intracellular retention and secretion behavior.

**Table 4:** **Overview of studies containing quantitative data excluded from the meta-analysis for the secretion outcome.** Shown are the shape of the curve, the Inverted curve, and the Decrease in Ymax, Reason for exclusion from meta-analysis. Total of 12 patients.

Author and Year	Shape of curve	Inverted curve	Decrease in Ymax	Reason for exclusion from meta-analysis
Barsh, 1981	Linear	No	Yes	Limited datapoint collection results in linear curve appearance
Steinmann, 1984	Linear	No	Yes	Limited datapoint collection results in a linear curve appearance
Bonaventure, 1986	Hyperbolic	No	Yes	Hyperbolic curve instead of the expected sigmoidal curve and curve doesn't start at zero
Wenstrup, 1986	Linear	No	Yes	Limited datapoint collection, result in linear curve appearance
Royce, 1988	sigmodal for control, linear for patient	No	Yes	The patient curve is linear compared to the sigmoidal curve of the control
Forlino, 1994	mixed linear	Yes	Yes	Unable to fit points to the curve due to random changes in direction
Besio, 2018	Hyperbolic	No	No	Hyperbolic curve instead of the expected sigmoidal curve

**Fig. 3:**
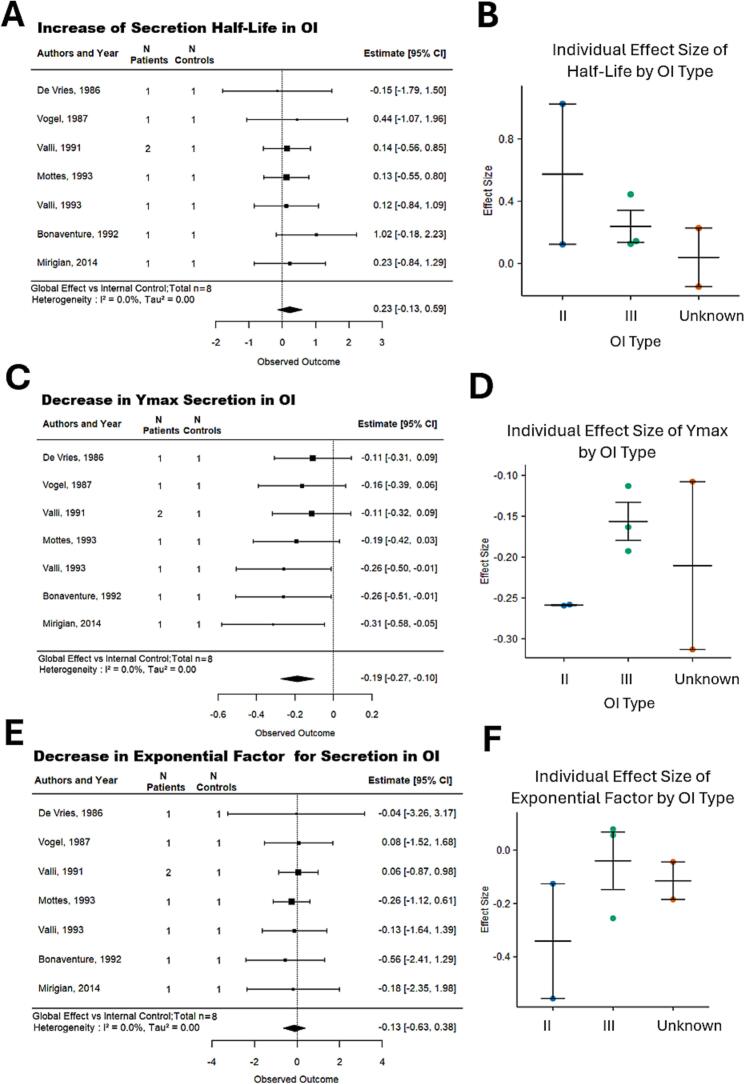
**Collagen secretion of OI patients compared to controls.** (A,C,E) Forest plots of percentage difference in OI collagen-I secretion half-life in OI collagen (A) Ymax Secretion (C) exponential factor (E) compared to control; positive values indicate higher levels in patients. Squares with lines depict the study-level effect, normalized mean difference with 95 % Cl; the size of the square is proportional to the study weight. The diamond represents the global effect size. Heterogeneity statistics I^2^ and τau^2^ are reported. (B, D, F) Individual patient effect size separated by OI type, collagen-I secretion half-life in OI collagen (B) Ymax Secretion (D) exponential factor (F) compared to control; positive values indicate higher levels in patients. Unknown are samples in which an OI type was not reported. Shown are individual data effect size points and their mean ± SEM.

The meta-analysis of changes in secretion half-life in OI collagen included 7 studies describing 8 patients, and showed an increase compared to controls, with a global effect size of 0.23 [95% CI: −0.13, 0.59] **(**Fig. 3A**)**. Heterogeneity was zero, with τau^2^ = 0.00 and I2 = 0.0%. The meta-analysis of the change in secretion Ymax in OI collagen showed a decrease compared to controls, with a global effect size of −0.19 [95% CI: −0.27, 0.10] **(**Fig. 3C**)**. Heterogeneity was zero, with τau^2^ of 0.00 and I^2^ of 0.0%. The meta-analysis of changes in the exponential secretion factor in OI collagen showed a decrease compared to controls, with a global effect size of −0.13 [95% CI: −0.63, 0.38] **(**Fig. 3E**)**. Heterogeneity was zero, with τau^2^ of 0.00 and I^2^ of 0.0%. The individual data subgroup by OI type for half-life, Ymax and exponential did not show any association with OI severity **(**Fig. 3B, D, F**)**. OI severity analysis is severely underpowered due to the lack of data, as only 5 of the 8 patients have their OI type reported in this outcome. All studies reporting collagen secretion were small, which did not allow to establish an association with base change mutation or the position of the mutation.

### 3.3 Crosslinking

**Fig. 4:**
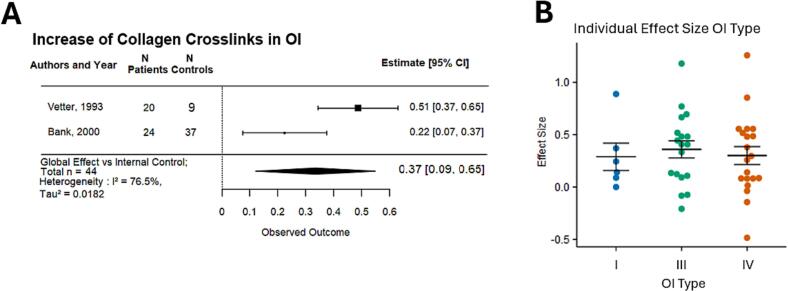
**Collagen crosslinks of OI patients compared to controls.** (A) Forest plot of percentage difference in collagen crosslink in OI, positive values indicate higher levels in patients. For A, squares with lines depict the study-level effect, normalized mean difference with 95 % Cl; the size of the square is proportional to the study weight. The diamond represents the global effect size. Heterogeneity statistics I^2^ and τau^2^ are reported. (B) Collagen crosslink in individual patient data separated by OI type. For B, shown are individual effect size data points and their mean ± SEM.

Crosslinks are small molecules that are formed between the telopeptide of one tropocollagen with the helical part of a neighboring tropocollagen (Robins, 2007). The crosslinks are measured by quantification of Hydroxylysylpyridinoline (HP), which is formed by a hydroxylysine residue, and lysylpyridinoline (LP), which is formed by a lysine residue (Vetter et al., 1993; Bank et al., 2000). Crosslinks were measured using reversed-phased high-performance liquid chromatography and the data is presented as HP + LP. The meta-analysis on the change in collagen-I crosslink in OI included 2 studies with 44 patients and demonstrated an increase compared to controls, with a global effect of 0.37 [95% CI: 0.02, 0.65] (Fig. 4A). The heterogeneity was unique with as was a τau^2^ of 0.02, and I^2^ of 76.5%, which is due to having only two studies with some difference in effect size. The individual data subgroup analysis by OI type shows no association with OI severity (Fig. 4B). The OI types I, III and IV are represented in this analysis with more than 5 patients per group. The data suggest that the increase in crosslinking is similar across all OI types, and the increase in lysine hydroxylation in OI is weakly associated with disease severity (Patel et al., 2026). Considering these two facts, an increase in crosslinking should be observed across OI type. Although evidence is limited to two publications, both studies included more than 20 patients. Collectively, these findings suggest that individuals with OI may exhibit higher collagen cross-link levels than healthy counterparts.

### 3.4 Fibril diameter

**Fig. 5:**
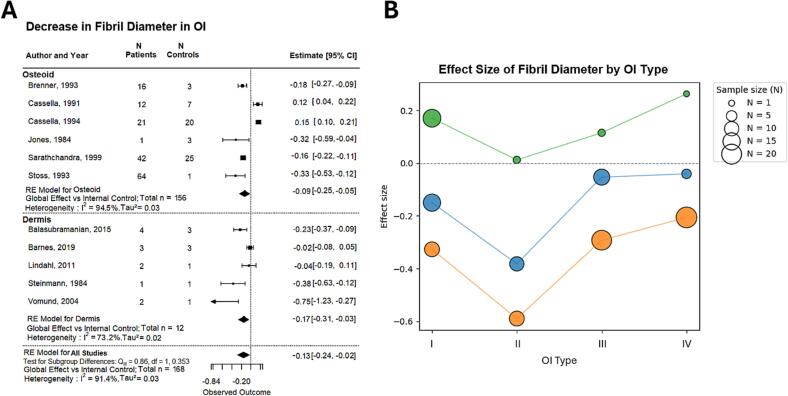
**Collagen diameter of OI patients compared to controls.** (A) Forest plot of percentage difference in collagen diameter in OI, negative values indicate lower levels in patients. Subgroup analysis is performed by the collagen sample. For A, squares with lines depict the study-level effect, normalized mean difference with 95 % Cls; the size of the square is proportional to the study weight. The diamond represents the global effect size. Heterogeneity statistics I^2^ and τau^2^ are reported. (B) Collagen diameter grouped by OI type within individual publication (each color represents a single publication).

Ultrathin sections of the bone sample were made, and then the fibril diameter was measured by transmission electron microscopy (Cassella et al., 1994). Data are presented in tabular form. The meta-analysis of changes in collagen fibril diameter included data for 156 patients for the osteoid measurements and 12 patients for the dermis measurements. Overall, fibril diameter in OI showed a decrease compared to control, with a global effect of −0.13 [95% CI: −0.24, −0.02] **(**Fig. 5A**)**. Heterogeneity was zero, with τau^2^ of 0.03 and I^2^ of 91.4%. When considering subgroup analysis, the osteoid had an effect size of −0.09 [95% CI: −0.25, −0.05], and the dermis subgroup had an effect of −0.17 [95% CI: - 0.31, −0.03]; the difference was non-significant, with a Q_M_ value of 0.86, no study reported data from both samples. Fibril diameter outcomes did not have individual patient data, however several studies provided aggregate grouping by OI type. Fig. 5**B** shows the effect size by OI type comparison within study (Cassella et al. represented in green, Sarathchandra et al. represented in blue and Stoss et al. represented in orange) (Cassella et al., 1994; Stöss and Freisinger, 1993; Sarathchandra et al., 1999). All three studies demonstrated the same relationship between OI types, where the fiber diameter was the smallest in OI type II, and similar in OI type I, III and IV. The three studies differed by a magnitude factor, meaning the Cassella et al. study has the highest value of each OI type and Stoss et al. study is the lowest for each OI type. The studies presented included diameter measurements on different bone samples (iliac crest and femur), which fundamentally affects the results (Berillis et al., 2006). Thus, while the primary studies reported noticeable variability in the direction of the effect, overall analysis suggests small decreases in OI collagen-I fibril diameter in both osteoid and dermis.

### 3.5 Melting temperature

The qualitative dataset for melting temperature comprises 30 studies with 31 patients (Table 5). These studies performed the collagen melting temperature assay described by Prockop (Bruckner and Prockop, 1981). The final data is a gel with collagen bands at different temperature points. It is unfeasible to determine at which exact temperature the collagen band loses 50% of its opacity. In addition, some publications state a melting temperature, but this value doesn't reflect an eye test. Another issue with gel is variable band sizes and faintness across the temperature interval, rather than a steady decrease. For the aforementioned reasons, gel data on melting temperature are considered only qualitatively. All studies report a decrease in melting temperature compared to the control.

**Table 5:** **Overview of studies containing qualitative data for the melting temperature outcome.** Shown are the number of patients (NP) and controls (NC), OI type or reported severity, patients' age (in years, y) and sex (male (M), female (F), not reported (NR)), data type and lower temperature. Total of 30 patients.

Author and Year	NP	NC	OI Type	Age (y)	Sex	Data type	Lower Temperature
Wenstrup, 1986	1	1	3	8	M	Gel	Yes
Wenstrup, 1986	1	1	4	1	M	Gel	Yes
Willing, 1988	1	1	2	infant	NR	Gel	Yes
Byers, 1988	1	1	2	infant	NR	Gel	Yes
Tenni, 1988	1	1	3	2	M	Gel	Yes
Royce, 1988	1	1	2	infant	F	Gel	Yes
Bateman, 1988	1	1	3	NR	NR	Gel	Yes
Superti-Furga, 1988	1	1	3	infant	M	Gel	Yes
Wenstrup, 1988	1	1	1	42	F	Gel	Yes
Pack, 1989	1	1	3	5	M	Gel	Yes
Starman, 1989	1	1	1	21	F	Gel	Yes
Wallis, 1990	1	1	2	infant	NR	Gel	Yes
Grange, 1990	1	1	2	infant	NR	Gel	Yes
Westerhausen, 1990	2	1	2	infant	NR	Gel	Yes
Deak, 1991	1	1	1	5	M	Gel	Yes
Steinmann, 1991	1	1	2	infant	F	Gel	Yes
Tenni, 1991	1	1	1	2	M	Gel	Yes
Tsuneyoshi, 1991	1	1	2	infant	NR	Gel	Yes
Wallis, 1992	1	1	2	infant	M	Gel	Yes
Fertala, 1993	1	1	2	infant	NR	Gel	Yes
Mottes, 1993	1	1	2	infant	F	Gel	Yes
Superti-Furga, 1993	1	1	1	5	M	Gel	Yes
Valli, 1993	1	1	2	infant	F	Gel	Yes
Chessler, 1993	1	1	2	infant	F	Gel	Yes
Zhuang, 1993	1	1	3	18	F	Gel	Yes
Bateman, 1994	1	1	4	NR	NR	Gel	Yes
Lightfoot, 1994	1	1	4	NR	NR	Gel	Yes
Pace, 2001	1	1	1	NR	M	Gel	Yes
Pace, 2001	1	1	2	infant	F	Gel	Yes
Galika, 2003	1	1	2	infant	NR	Gel	Yes

**Fig. 6:**
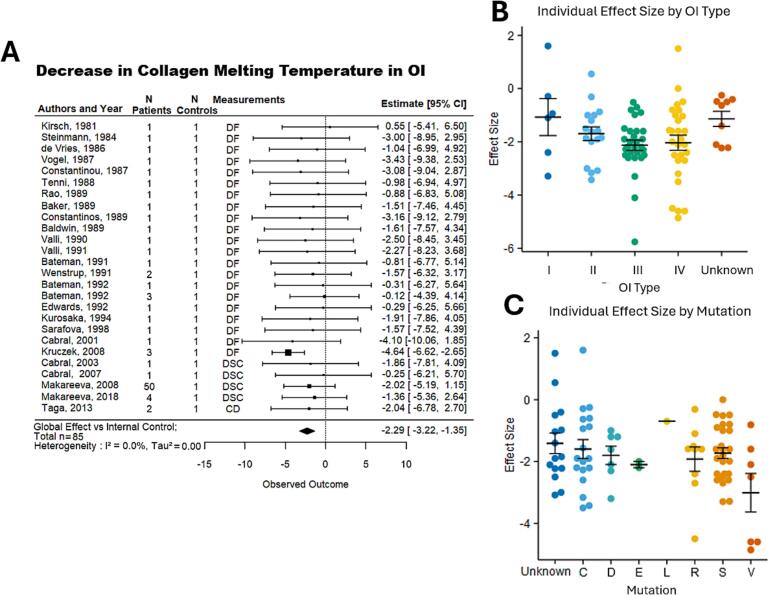
**Collagen melting temperature of OI patients compared to controls.** (A) Forest plot of raw difference in collagen melting temperature in OI, negative values indicate lower levels in patients. For A, squares with lines depict the study-level effect, raw difference with 95 % Cl; the size of the square is proportional to the study weight. The diamond represents the global effect size. Heterogeneity statistics I^2^ and τau^2^ are reported. (B) Collagen melting temperature effect size for individual patient data separated by OI type, Unknown are samples in which OI type is unclear. For B, shown are individual effect size datapoints and their mean ± SEM. (C) Collagen melting temperature effect size for individual patient data separated by amino acid mutation. Shown are individual effect size datapoints and their mean ± SEM. For the X-axis labels, C is cysteine, D is aspartic acid, E is glutamic acid, L is leucine, R is arginine, S is serine, and V is valine.

For meta-analysis data, the Prockop method was used with different instrument measurements. Densitometry was used to measure the signal [^3^H] proline of the gel. When a gel was not used, the signal was assessed by differential scanning calorimetry or circular dichroism. The important limitation of the Prockop method is that it includes a digestion step, which fundamentally alters the denaturation process and introduces variability into the assay. The meta-analysis of changes in collagen melting temperature in OI included 26 studies describing 85 patients and showed a significant decrease compared to control −2.29 [95% CI: −3.22, −1.35] **(**Fig. 6A**)**. Heterogeneity was zero, with tau2 of 0.00 and I2 of 0.0%. The individual data subgroup by OI type for melting temperature showed no association with OI severity **(**Fig. 6B**)**. Consistent with the analysis performed in the largest study with 47 OI patients (Makareeva et al., 2008), of we did not observe association between the base change mutation and change in melting temperature **(**Fig. 6C**)**.

## 4 Discussion

This systematic review and meta-analysis compiled and quantified all available biological data on collagen-I folding, secretion, crosslinks, fibril diameter and melting temperature in individuals with OI. Using the knowledge synthesis methodology allowed us to combine the data for 327 patients, a substantial sample size for a rare disease. We identified difference between OI and its healthy counterpart in collagen-I folding, secretion, crosslinks, fibril diameter and melting temperature. Collagen-I crosslink and melting temperature were associated with disease severity, and fibril diameter was more affected in the most severe OI Type II. Taken together, these findings present the first quantitative consensus on selected outcomes, which help to generate new hypotheses regarding the causal relationships among outcomes.

Our study demonstrates that OI collagen-I demonstrates significantly slower folding and reduced secretion. OI collagen-I folding and secretion could potentially be affected by the post-translational modification (PTM) levels and ER functionality (ER stress). OI collagen was shown to have increased PTM levels (Patel et al., 2026). However, no study to date has investigated OI collagen-I PTM level alongside collagen folding kinetics; therefore, it is difficult to determine the causative relationship between these processes. The ER stress was demonstrated in OI by previous microscopy studies showing enlarged cisternae and recent studies that measured high expression of ER stress markers (Besio et al., 2019; Besio et al., 2018b; Stöß et al., 1986). Recent studies have investigated the administration of 4-phenyl butyric acid (4PBA) in OI cultured osteoblasts, which improved collagen secretion in OI (Takeyari et al., 2021). Both mouse and zebrafish models of OI have shown higher BMD and BV/TV with 4PBA administration, suggesting that limited collagen-I secretion contributes to poor bone parameters in OI (Scheiber et al., 2022; Gioia et al., 2017). Additionally, Takeyari et al. showed that the administration of 4BPA did not affect the collagen-I PTM levels, which suggests ER stress levels may not contribute to the slower collagen-I folding (Takeyari et al., 2021). However, it is unclear whether slower collagen folding contributes to ER stress in collagen. Taken together, this suggests that there are two collagen-retention events: the first is slower folding kinetics, and the second is retention of the fully folded collagen.

At the fibril level, both collagen crosslinks and fibril diameter appear to be compromised. The increase in total crosslinks contributes to fibril stiffness, which reduces the capacity of the collagen matrix to dissipate energy during deformation (Saito and Marumo, 2015). Since elasticity is the mechanical role of collagen fibrils, the increased rigidity was proposed to be a significant driver of bone brittleness (Saito and Marumo, 2015; Zimmermann et al., 2011). Recent studies in the field of crosslinks have been focused on advance glycation endproducts (AGE); however, to our knowledge AGE has not been yet reported in OI. Fibril diameters are intrinsically linked to biomechanical strength. Consequently, the smaller diameters observed in OI likely compromise the tissue overall load-bearing capacity (Yamamoto and Nakamura, 2017). Additionally, polarized light microscopy and second-harmonic generation imaging reveal that the fibrils are organized disorderedly compared to healthy controls (LaComb et al., 2008). We also demonstrated that collagen melting temperatures are consistently lower in OI. However, this biochemical measurement is not well-linked to mechanical stability, but rather reflects thermal and enzymatic denaturation under non-physiological conditions (Bruckner and Prockop, 1981). Therefore, changes in melting temperature should be viewed as complementary evidence of collagen-I alteration, rather than a mechanistic link to bone brittleness. Taken together, collagen-I alterations at multiple hierarchical levels likely contribute to bone fragility in OI.

Several limitations must be acknowledged in this study. First, a significant portion of the literature included here dates to an era when diagnostic resources for Osteogenesis Imperfecta were less sophisticated and not as widely accessible in clinical practice. Consequently, inconsistencies in disease classification may have arisen, potentially introducing bias into the severity analysis. Furthermore, non-collagen-I forms of OI, which have only been well-characterized in more recent years, may have been inadvertently included in earlier OI studies. However, given the prevalence of mutation underlying OI (85–90% of OI cases attributed to COL1A1 or COL1A2 variants (Jovanovic and Marini, 2024)), the potential contribution of non-collagen OI forms is likely low. Even when genetic diagnosis was performed, the underlying mutation (base change or frameshift mutation) was rarely reported, preventing genotype-association analysis with the selected outcomes. Additionally, the absence of demographic data, including age and sex, limited our capacity to explore these variables as potential modifiers of the measured outcomes. It is important to note that the parameters used to characterize folding and secretion curves, including half-time, Ymax, and exponential factor, were used as descriptive metrics of the observed curves, and do not fully capture mechanistic features of underlying processes. Due to these limitations, the small sample sizes and heterogeneity across studies, the quantitative estimates generated by this meta-analysis should be interpreted cautiously rather than as definitive effect magnitudes. Instead, the findings are most useful for identifying general trends that align with current biological understanding, describing broad associations with disease severity, and highlighting areas where data and characterization remain limited.

Despite these constraints, the descriptive quantitative synthesis provided by our study represents the most extensive synthesis to date of biological data regarding OI collagen-I folding, secretion, crosslinking, fibril diameter, and melting temperature. By aggregating data from a cohort of 329 OI patients, we achieved a substantial sample size for a rare disease, providing novel insights into the pathophysiology of OI. These findings should also be interpreted within the broader context of the collagen pathway. While a definitive causal relationship between the selected outcomes has not yet been established, our findings provide a critical framework for future investigations into how intracellular collagen defects and extracellular matrix processing together contribute to skeletal fragility. Finally, these results offer several meaningful translational implications for the field.

## CRediT authorship contribution statement

**Priyesh Patel:** Writing – review & editing, Writing – original draft, Visualization, Methodology, Investigation, Formal analysis, Data curation, Conceptualization. **Sirion Aksornthong:** Writing – review & editing, Data curation. **Svetlana V. Komarova:** Writing – review & editing, Supervision, Investigation, Funding acquisition, Conceptualization.

## Authors contributions

PP conceived the study, developed the search strategy, performed screening and data extraction, participated in development of data analysis protocols and performed the data analysis, wrote the first draft of the manuscript. SA performed screening, participated in development of data analysis protocols and edited the manuscript. SVK conceived the study, obtained funding, developed the data analysis protocols, participated in data analysis and edited the manuscript. All authors read and approved the final manuscript.

## Funding

This study was supported by the developmental grant (#87210-CAN-24) from the Shriners of North America and by 10.13039/501100000038Natural Sciences and Engineering Research Council of Canada (NSERC RGPIN-288253).

## Declaration of competing interest

The authors declare the following financial interests/personal relationships which may be considered as potential competing interests: Svetlana Komarova reports financial support was provided by SHRINERS HOSP FOR CHILDREN. If there are other authors, they declare that they have no known competing financial interests or personal relationships that could have appeared to influence the work reported in this paper.

## Data Availability

Data will be made available on request.

## References

[bb0005] Bank R.A., Tekoppele J.M., Janus G.J.M., Wassen M.H.M., Pruijs H.E.H., van der Sluijs H.A.H., Sakkers R.J.B. (2000). Pyridinium cross-links in bone of patients with osteogenesis imperfecta: evidence of a normal intrafibrillar collagen packing. J. Bone Miner. Res..

[bb0010] Berillis P., Emfietzoglou D., Tzaphlidou M. (2006). Collagen fibril diameter in relation to bone site and to calcium/phosphorus ratio. Sci. World J..

[bb0015] Besio R., Iula G., Garibaldi N., Cipolla L., Sabbioneda S., Biggiogera M., Marini J.C., Rossi A., Forlino A. (2018). 4-PBA ameliorates cellular homeostasis in fibroblasts from osteogenesis imperfecta patients by enhancing autophagy and stimulating protein secretion. Biochim. Biophys. Acta (BBA) - Mol. Basis Dis..

[bb0020] Besio R., Iula G., Garibaldi N., Cipolla L., Sabbioneda S., Biggiogera M., Marini J.C., Rossi A., Forlino A. (2018). 4-PBA ameliorates cellular homeostasis in fibroblasts from osteogenesis imperfecta patients by enhancing autophagy and stimulating protein secretion. Biochim. Biophys. Acta (BBA) - Mol. Basis Dis..

[bb0025] Besio R., Garibaldi N., Leoni L., Cipolla L., Sabbioneda S., Biggiogera M., Mottes M., Aglan M., Otaify G.A., Temtamy S.A. (2019). Cellular stress due to impairment of collagen prolyl hydroxylation complex is rescued by the chaperone 4-phenylbutyrate. Dis. Model. Mech..

[bb0030] Bruckner P., Prockop D.J. (1981). Proteolytic enzymes as probes for the triple-helical conformation of procollagen. Anal. Biochem..

[bb0035] Buss D.J., Kröger R., McKee M.D., Reznikov N. (2022). Hierarchical organization of bone in three dimensions: a twist of twists. Journal of Structural Biology: X.

[bb0040] Byers P.H., Starman B.J., Cohn D.H., Horwitz A.L. (1988). A novel mutation causes a perinatal lethal form of osteogenesis imperfecta. An insertion in one alpha 1(I) collagen allele (COL1A1). J. Biol. Chem..

[bb0045] Cassella J.P., Barber P., Catterall A.C., Ali S.Y. (1994). A morphometric analysis of osteoid collagen fibril diameter in osteogenesis imperfecta. Bone.

[bb0050] de Vries W.N., de Wet W.J. (1986). The molecular defect in an autosomal dominant form of osteogenesis imperfecta. Synthesis of type I procollagen containing cysteine in the triple-helical domain of pro-alpha 1(I) chains. J. Biol. Chem..

[bb0055] Engler J.B. (2025). Tidyplots empowers life scientists with easy code-based data visualization. iMeta.

[bb0060] Furukawa T.A., Barbui C., Cipriani A., Brambilla P., Watanabe N. (2006). Imputing missing standard deviations in meta-analyses can provide accurate results. J. Clin. Epidemiol..

[bb0065] Garnero P. (2015). The role of collagen organization on the properties of bone. Calcif. Tissue Int..

[bb0070] Gioia R., Tonelli F., Ceppi I., Biggiogera M., Leikin S., Fisher S., Tenedini E., Yorgan T.A., Schinke T., Tian K. (2017). The chaperone activity of 4PBA ameliorates the skeletal phenotype of Chihuahua, a zebrafish model for dominant osteogenesis imperfecta. Hum. Mol. Genet..

[bb0075] Hunter J.D. (2007). Matplotlib: a 2D graphics environment. Computing in Science & Engineering.

[bb0080] Jovanovic M., Marini J.C. (2024). Update on the genetics of osteogenesis imperfecta. Calcif. Tissue Int..

[bb0085] LaComb R., Nadiarnykh O., Campagnola P.J. (2008). Quantitative second harmonic generation imaging of the diseased state osteogenesis imperfecta: experiment and simulation. Biophys. J..

[bb0090] Lambert P.C., Sutton A.J., Abrams K.R., Jones D.R. (2002). A comparison of summary patient-level covariates in meta-regression with individual patient data meta-analysis. J. Clin. Epidemiol..

[bb0095] Makareeva E., Mertz E.L., Kuznetsova N.V., Sutter M.B., DeRidder A.M., Cabral W.A., Barnes A.M., McBride D.J., Marini J.C., Leikin S. (2008). Structural heterogeneity of type I collagen triple Helix and its role in osteogenesis imperfecta *. J. Biol. Chem..

[bb0100] Makareeva E., Aviles N.A., Leikin S. (2011). Chaperoning osteogenesis: new protein-folding disease paradigms. Trends Cell Biol..

[bb0105] Malhotra V., Erlmann P. (2015). The pathway of collagen secretion. Annu. Rev. Cell Dev. Biol..

[bb0110] Mikolajewicz N., Komarova S.V. (2019). Meta-analytic methodology for basic research: a practical guide. Front. Physiol..

[bb0115] Mirigian L.S., Makareeva E., Leikin S. (2014). Pulse-chase analysis of procollagen biosynthesis by azidohomoalanine labeling. Connect. Tissue Res..

[bb0120] Nijhuis W.H., Eastwood D.M., Allgrove J., Hvid I., Weinans H.H., Bank R.A., Sakkers R.J. (2019). Current concepts in osteogenesis imperfecta: bone structure, biomechanics and medical management. J. Child. Orthop..

[bb0125] Ouzzani M., Hammady H., Fedorowicz Z., Elmagarmid A. (2016). Rayyan—a web and mobile app for systematic reviews. Syst. Rev..

[bb0130] Page M.J., McKenzie J.E., Bossuyt P.M., Boutron I., Hoffmann T.C., Mulrow C.D., Shamseer L., Tetzlaff J.M., Akl E.A., Brennan S.E. (2021). The PRISMA 2020 statement: an updated guideline for reporting systematic reviews. BMJ.

[bb0135] Patel P., Aksornthong S., Komarova S.V. (2026). Post-translational modifications of collagen type I in osteogenesis imperfecta: systematic review and meta-analysis. Bone Reports.

[bb0140] Raghunath M., Bruckner P., Steinmann B. (1994). Delayed triple Helix formation of mutant collagen from patient with osteogenesis imperfecta. J. Mol. Biol..

[bb0145] Rauch F., Glorieux F.H. (2004). Osteogenesis imperfecta. Lancet.

[bb0150] Revell C.K., Jensen O.E., Shearer T., Lu Y., Holmes D.F., Kadler K.E. (2021). Collagen fibril assembly: new approaches to unanswered questions. Matrix Biol. Plus.

[bb0155] Riley R.D., Higgins J.P.T., Deeks J.J. (2011). Interpretation of random effects meta-analyses. BMJ.

[bb0160] Robins S.P. (2007). Biochemistry and functional significance of collagen cross-linking. Biochem. Soc. Trans..

[bb0165] Saito M., Marumo K. (2015). Effects of collagen crosslinking on bone material properties in health and disease. Calcif. Tissue Int..

[bb0170] Sarathchandra P., Pope F.M., Ali S.Y. (1999). Morphometric analysis of type I collagen fibrils in the osteoid of osteogenesis imperfecta. Calcif. Tissue Int..

[bb0175] Scheiber A.L., Wilkinson K.J., Suzuki A., Enomoto-Iwamoto M., Kaito T., Cheah K.S.E., Iwamoto M., Leikin S., Otsuru S. (2022). 4PBA reduces growth deficiency in Osteogenesis Imperfecta by enhancing transition of hypertrophic chondrocytes to osteoblasts. JCI Insight.

[bb0180] Seo M., White I.R., Furukawa T.A., Imai H., Valgimigli M., Egger M., Zwahlen M., Efthimiou O. (2021). Comparing methods for estimating patient-specific treatment effects in individual patient data meta-analysis. Stat. Med..

[bb0185] Stöss H., Freisinger P. (1993). Collagen fibrils of osteoid in osteogenesis imperfecta: morphometrical analysis of the fibril diameter. Am. J. Med. Genet..

[bb0190] Stöß H., Pontz B.F., Pesch H.J., Ott R. (1986). Heterogeneity of osteogenesis imperfecta. Biochemical and morphological findings in a case of type III according to Sillence. Eur. J. Pediatr..

[bb0195] Takeyari S., Kubota T., Ohata Y., Fujiwara M., Kitaoka T., Taga Y., Mizuno K., Ozono K. (2021). 4-Phenylbutyric acid enhances the mineralization of Osteogenesis Imperfecta iPSC-derived osteoblasts. J. Biol. Chem..

[bb0200] Tauer J.T., Robinson M.-E., Rauch F. (2019). Osteogenesis imperfecta: new perspectives from clinical and translational research. JBMR. Plus.

[bb0205] Tournis S., Dede A.D. (2018). Osteogenesis imperfecta - a clinical update. Metabolism.

[bb0210] Valli M., Sangalli A., Rossi A., Mottes M., Forlino A., Tenni R., Pignatti P.F., Cetta G. (1993). Osteogenesis imprefecta and type-I collagen mutations. Eur. J. Biochem..

[bb0215] Van Dijk F.S., Pals G., Van Rijn R.R., Nikkels P.G.J., Cobben J.M. (2010). Classification of osteogenesis imperfecta revisited. Eur. J. Med. Genet..

[bb0220] Vetter U., Weis M.A., Mörike M., Eanes E.D., Eyre D.R. (1993). Collagen crosslinks and mineral crystallinity in bone of patients with osteogenesis imperfecta. J. Bone Miner. Res..

[bb0225] Viechtbauer W. (2010). Conducting meta-analyses in R with the metafor package. J. Stat. Softw..

[bb0230] Viechtbauer W., Cheung M.W.-L. (2010). Outlier and influence diagnostics for meta-analysis. Res. Synth. Methods.

[bb0235] Wallis G.A., Starman B.J., Schwartz M.F., Byers P.H. (1990). Substitution of arginine for glycine at position 847 in the triple-helical domain of the alpha 1 (I) chain of type I collagen produces lethal osteogenesis imperfecta. Molecules that contain one or two abnormal chains differ in stability and secretion. J. Biol. Chem..

[bb0240] Yamamoto N., Nakamura S. (2017). Relationships between the tensile strength and diameter of collagen fibrils isolated from mouse tail tendons. J. Biomech. Sci. Eng..

[bb0245] Zimmermann E.A., Schaible E., Bale H., Barth H.D., Tang S.Y., Reichert P., Busse B., Alliston T., Ager J.W., Ritchie R.O. (2011). Age-related changes in the plasticity and toughness of human cortical bone at multiple length scales. Proc. Natl. Acad. Sci. USA.

