# Supplementary material for: Collagen secretion and maturation in osteogenesis imperfecta: Systematic review and meta-analysis
Source: Bone Rep. 2026 Jun 8;30:101928. doi: 10.1016/j.bonr.2026.101928 (PMC13311377; doi:10.1016/j.bonr.2026.101928)
Supplement: Supplementary file 1 — Supplementary material [file mmc1.pdf]

Supplementary material for the manuscript "Collagen secretion and maturation in osteogenesis imperfecta: systematic review and meta-analysis" by P. Patel, S. Aksornthong, and SV Komarova

Supplementary Method 1: Search Strategy

Example of search strategy applied for web of Science database

1 = (TS = (osteogenesis imperfecta))

2 = (TS = (brittle bone disease))

3 = (TS = (brittle bone dysplasia))

4 = (TS = (collagen))

5 = (TS = (Collagen folding, folding kinetics, protein folding, triple helix assembly))

6 = (TS = (Collagen secretion, protein transport, tango1))

7 = (TS = (Collagen crosslinks, hydroxylysylpyridinoline, lysylpyridinoline))

8 = (TS = (fibril diameter, fibril analysis, bone cross-section, osteoid, dermis))

9 = (TS = (melting temperature, collagen stability, differential scanning calorimetry, circular dichroism))

10 = #1 OR #2 OR #3

11 = #4 OR #5 OR #6 OR #7 OR #8 OR #9

13 = #10 AND #12

Supplementary Method 2: Quality checklist

S2.1 Description of the checklist for quality assessment for clinical study

1. Was the OI type specified. (0.5 given for qualitative description, 1pts where type 2 can be inferred by perinatal lethal description, 1.5pts for type description based off silence classification, 2pts for type description and mutation)
2. Age description of patient (1 pts)
3. Sex description of patient (1 pts)
4. Did the study have age-matched control (1pts)
5. Was the experimental method described (1 pts)
6. Did they run triplicates (0.5 for duplicates, 1 pts for triplicates)
7. Was the method used the standard method (1 pts)
8. Did the patient pool have different types of OI (1pts)
9. Is the sample size larger than 5 (1 pts)
10. Does the study provide individual patient data (1pts)

Supplementary Table 1. PRISMA checklist

Section and Topic	Item #	Checklist item	Location where item is reported
**TITLE**			
Title	1	Identify the report as a systematic review.	Title and abstract
**ABSTRACT**			
Abstract	2	See the PRISMA 2020 for Abstracts checklist.	Material and Methods paragraph 1
**INTRODUCTION**			
Rationale	3	Describe the rationale for the review in the context of existing knowledge.	Introduction paragraph 2
Objectives	4	Provide an explicit statement of the objective(s) or question(s) the review addresses.	Introduction paragraph 3
**METHODS**			
Eligibility criteria	5	Specify the inclusion and exclusion criteria for the review and how studies were grouped for the syntheses.	Material and Methods Information sources, search strategy, eligibility criteria, and screening
Information sources	6	Specify all databases, registers, websites, organisations, reference lists and other sources searched or consulted to identify studies. Specify the date when each source was last searched or consulted.	Material and Methods Information sources, search strategy, eligibility criteria, and screening
Search strategy	7	Present the full search strategies for all databases, registers and websites, including any filters and limits used.	Material and Methods Information sources, search strategy, eligibility criteria, and screening
Selection process	8	Specify the methods used to decide whether a study met the inclusion criteria of the review, including how many reviewers screened each record and each report retrieved, whether they worked independently, and if applicable, details of automation tools used in the process.	Material and Methods Information sources, search strategy, eligibility criteria, and screening
Data collection process	9	Specify the methods used to collect data from reports, including how many reviewers collected data from each report, whether they worked independently, any processes for obtaining or confirming data from study investigators, and if applicable, details of automation tools used in the process.	Material and Methods Data extraction
Data items	10a	List and define all outcomes for which data were sought. Specify whether all results that were compatible with each outcome domain in each study were sought (e.g. for all measures, time points, analyses), and if not, the methods used to decide which results to collect.	Material and Methods Data extraction
	10b	List and define all other variables for which data were sought (e.g. participant and intervention characteristics, funding sources). Describe any assumptions made about any missing or unclear information.	Material and Methods Data extraction
Study risk of bias assessment	11	Specify the methods used to assess risk of bias in the included studies, including details of the tool(s) used, how many reviewers assessed each study and whether they worked independently, and if applicable, details of automation tools used in the process.	Material and Methods Assessment of bias, Supplementary Fig S2
Effect measures	12	Specify for each outcome the effect measure(s) (e.g. risk ratio, mean difference) used in the synthesis or presentation of results.	Material and Methods Study Level Outcomes
Synthesis methods	13a	Describe the processes used to decide which studies were	Material and Methods

Section and Topic	Item #	Checklist item	Location where item is reported
		eligible for each synthesis (e.g. tabulating the study intervention characteristics and comparing against the planned groups for each synthesis (item #5)).	Meta-Analysis
	13b	Describe any methods required to prepare the data for presentation or synthesis, such as handling of missing summary statistics, or data conversions.	Material and Methods<br>Study Level Outcomes<br>Meta-Analysis
	13c	Describe any methods used to tabulate or visually display results of individual studies and syntheses.	Material and Methods<br>Meta-Analysis
	13d	Describe any methods used to synthesize results and provide a rationale for the choice(s). If meta-analysis was performed, describe the model(s), method(s) to identify the presence and extent of statistical heterogeneity, and software package(s) used.	Material and Methods<br>Meta-Analysis
	13e	Describe any methods used to explore possible causes of heterogeneity among study results (e.g. subgroup analysis, meta-regression).	Material and Methods<br>Meta-Analysis
	13f	Describe any sensitivity analyses conducted to assess robustness of the synthesized results.	Material and Methods<br>Supplementary Fig. S2
Reporting bias assessment	14	Describe any methods used to assess risk of bias due to missing results in a synthesis (arising from reporting biases).	N/A
Certainty assessment	15	Describe any methods used to assess certainty (or confidence) in the body of evidence for an outcome.	N/A
**RESULTS**			
Study selection	16a	Describe the results of the search and selection process, from the number of records identified in the search to the number of studies included in the review, ideally using a flow diagram.	Figure 1
	16b	Cite studies that might appear to meet the inclusion criteria, but which were excluded, and explain why they were excluded.	Tables 2, 4
Study characteristics	17	Cite each included study and present its characteristics.	Tables 1, 3, 5
Risk of bias in studies	18	Present assessments of risk of bias for each included study.	Table 1
Results of individual studies	19	For all outcomes, present, for each study: (a) summary statistics for each group (where appropriate) and (b) an effect estimate and its precision (e.g. confidence/credible interval), ideally using structured tables or plots.	Figures 2-6
Results of syntheses	20a	For each synthesis, briefly summarise the characteristics and risk of bias among contributing studies.	Results, Figures 2-6<br>Supplementary Fig S2
	20b	Present results of all statistical syntheses conducted. If meta-analysis was done, present for each the summary estimate and its precision (e.g. confidence/credible interval) and measures of statistical heterogeneity. If comparing groups, describe the direction of the effect.	Results, Figures 2,3,4
	20c	Present results of all investigations of possible causes of heterogeneity among study results.	Figures 2-6
	20d	Present results of all sensitivity analyses conducted to assess the robustness of the synthesized results.	Supplementary Fig S2
Reporting biases	21	Present assessments of risk of bias due to missing results (arising from reporting biases) for each synthesis assessed.	Table 1, Supplementary Fig S2
Certainty of evidence	22	Present assessments of certainty (or confidence) in the body of evidence for each outcome assessed.	Results, Figures 2-6

Section and Topic	Item #	Checklist item	Location where item is reported
**DISCUSSION**			
Discussion	23a	Provide a general interpretation of the results in the context of other evidence.	Discussion paragraph 2,3,4
	23b	Discuss any limitations of the evidence included in the review.	Discussion paragraph 5
	23c	Discuss any limitations of the review processes used.	Discussion paragraph 5
	23d	Discuss implications of the results for practice, policy, and future research.	Discussion paragraph 6
**OTHER INFORMATION**			
Registration and protocol	24a	Provide registration information for the review, including register name and registration number, or state that the review was not registered.	Material and Methods paragraph 1
	24b	Indicate where the review protocol can be accessed, or state that a protocol was not prepared.	NA
	24c	Describe and explain any amendments to information provided at registration or in the protocol.	NA
Support	25	Describe sources of financial or non-financial support for the review, and the role of the funders or sponsors in the review.	Done according to Journal requirements
Competing interests	26	Declare any competing interests of review authors.	Done according to Journal requirements
Availability of data, code and other materials	27	Report which of the following are publicly available and where they can be found: template data collection forms; data extracted from included studies; data used for all analyses; analytic code; any other materials used in the review.	Done according to Journal requirements

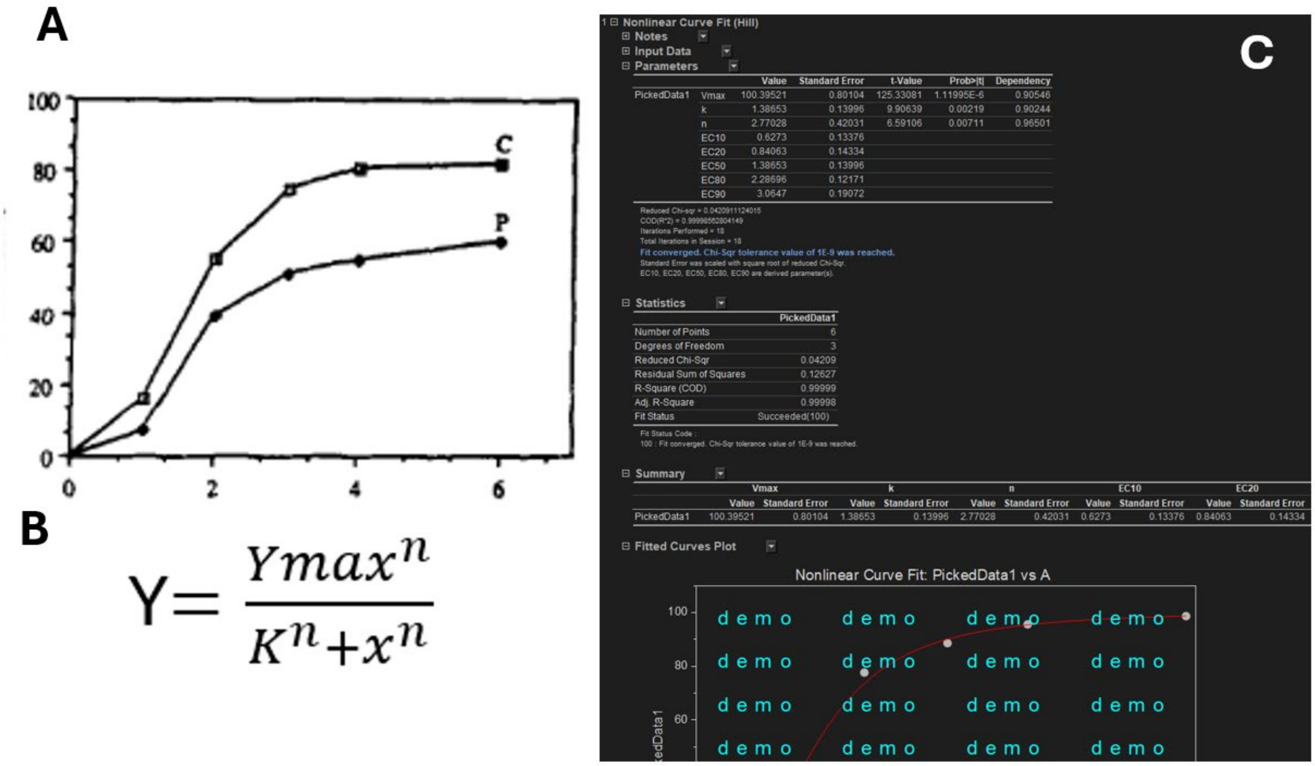

Supplementary Figure S1. Digitizing Curve process

A: This is the raw data (image) representing the secretion curve in Valli et al. The C curve is the control and the P curve is the patient. B: This is the equation that the OriginPro software is trying to fit individual data points into. Y represents the Y values, Ymax represents the maximum Y values, n is the exponential factor, and K is the half-life term. C: This is the output from the software once all the datapoints are picked.

Valli, M., et al., *A de novo G to T transversion in a pro-alpha 1 (I) collagen gene for a moderate case of osteogenesis imperfecta. Substitution of cysteine for glycine 178 in the triple helical domain*. Journal of Biological Chemistry, 1991. **266**(3): p. 1872-8.

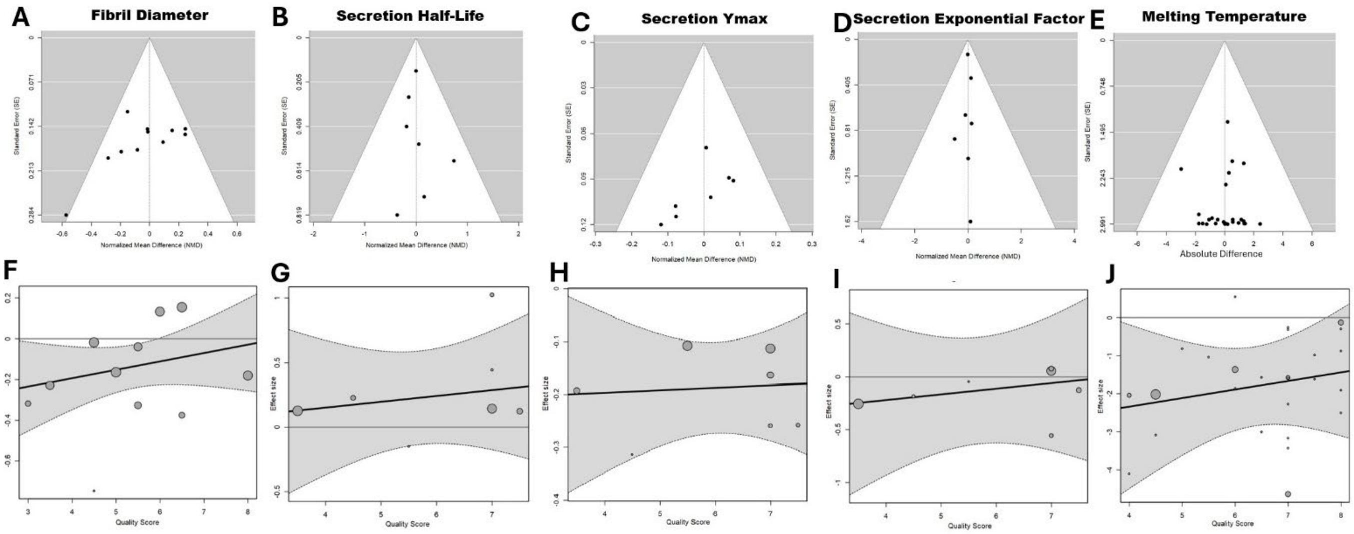

Supplementary Figure S2. Publication Bias Analysis. (A-E) Funnel plot for percentage change in Fibril Diameter (A), percentage change in Secretion halt-life (B), and percentage change in secretion Ymax (C), percentage change in secretion exponential factor (D), Absolute difference in melting temperature change. (F-J) Association of the effect size with quality score for fibril diameter (F), Secretion half-life(G), Secretion Ymax (H), Secretion exponential factor (I) and melting temperature (J). The analysis was performed using datasets reported on figures 3A, 3C, 3E for secretion outcome, 4A for fibril diameter, and 5A for melting temperature.

Supplementary bibliography

Reference for Qualitative data for secretion outcome

1. Valli, M., et al., *Osteogenesis imprefecta and type-I collagen mutations.* European Journal of Biochemistry, 1993. **211**(3): p. 415-419.
2. Barsh, G.S., K.E. David, and P.H. Byers, *Type I osteogenesis imperfecta: a nonfunctional allele for pro alpha 1 (I) chains of type I procollagen.* Proceedings of the National Academy of Sciences of the United States of America, 1982. **79**(12): p. 3838-42.
3. Wenstrup, R.J., P. Tsipouras, and P.H. Byers, *Osteogenesis imperfecta type IV. Biochemical confirmation of genetic linkage to the pro alpha 2(I) gene of type I collagen.* Journal of Clinical Investigation, 1986. **78**(6): p. 1449-55.
4. Byers, P.H., et al., *A novel mutation causes a perinatal lethal form of osteogenesis imperfecta. An insertion in one alpha 1(I) collagen allele (COL1A1).* Journal of Biological Chemistry, 1988. **263**(16): p. 7855-61.
5. Willing, M.C., et al., *Heterozygosity for a large deletion in the alpha 2(I) collagen gene has a dramatic effect on type I collagen secretion and produces perinatal lethal osteogenesis imperfecta.* Journal of Biological Chemistry, 1988. **263**(17): p. 8398-404.
6. Bateman, J.F., et al., *A frameshift mutation results in a truncated nonfunctional carboxyl-terminal pro alpha 1(I) propeptide of type I collagen in osteogenesis imperfecta.* Journal of Biological Chemistry, 1989. **264**(19): p. 10960-4.
7. Marini, J.C., et al., *Osteogenesis imperfecta type IV. Detection of a point mutation in one alpha 1(I) collagen allele (COL1A1) by RNA/RNA hybrid analysis.* J Biol Chem, 1989. **264**(20): p. 11893-900.
8. Wallis, G.A., et al., *Substitution of arginine for glycine at position 847 in the triple-helical domain of the alpha 1 (I) chain of type I collagen produces lethal osteogenesis imperfecta. Molecules that contain one or two abnormal chains differ in stability and secretion.* Journal of Biological Chemistry, 1990. **265**(30): p. 18628-33.
9. Bateman, J.F., et al., *Characterization of a type I collagen alpha 2(I) glycine-586 to valine substitution in osteogenesis imperfecta type IV. Detection of the mutation and prenatal diagnosis by a chemical cleavage method.* Biochemical Journal, 1991. **276**(Pt 3): p. 765-70.
10. Wallis, G.A., et al., *A tripeptide deletion in the triple-helical domain of the pro alpha 1(I) chain of type I procollagen in a patient with lethal osteogenesis imperfecta does not alter cleavage of the molecule by N-proteinase.* Journal of Biological Chemistry, 1992. **267**(35): p. 25529-34.
11. Bateman, J.F., et al., *Characterization of three osteogenesis imperfecta collagen alpha 1(I) glycine to serine mutations demonstrating a position-dependent gradient of phenotypic severity.* The Biochemical journal, 1992. **288**: p. 131-5.
12. Superti-Furga, A., et al., *An intronic deletion leading to skipping of exon 21 of COL1A2 in a boy with mild osteogenesis imperfecta.* Connective Tissue Research, 1993. **29**(1): p. 31-40.
13. Valli, M., et al., *Extracellular matrix deposition in cultured dermal fibroblasts from four probands affected by osteogenesis imperfecta.* Matrix, 1993. **13**(4): p. 275-80.
14. Constantinou-Deltas, C.D., R.L. Ladda, and D.J. Prockop, *Somatic cell mosaicism: another source of phenotypic heterogeneity in nuclear families with osteogenesis imperfecta.* Am J Med Genet, 1993. **45**(2): p. 246-51.
15. Mundlos, S., et al., *Multiexon Deletions in the Type I Collagen COL1A2 Gene in Osteogenesis Imperfecta Type: IB MOLECULES CONTAINING THE SHORTENED a2(I) CHAINS SHOW DIFFERENTIAL INCORPORATION INTO THE BONE AND SKIN EXTRACELLULAR MATRIX* Journal of Biological Chemistry, 1996. **271**(35): p. 21068-21074.

16. Nicholls, A.C., et al., *Splice site mutation causing deletion of exon 21 sequences from the proα2(I) chain of type I collagen in a patient with severe dentinogenesis imperfecta but very mild osteogenesis imperfecta*. Human Mutation, 1996. **7**(3): p. 219-227.
17. Oliver, J.E., et al., *Mutation in the carboxy-terminal propeptide of the proα1 (I) chain of type I collagen in a child with severe osteogenesis imperfecta (OI type III): Possible implications for protein folding*. Human Mutation, 1996. **7**(4): p. 318-326.
18. Pace, J.M., et al., *Deletions and duplications of Gly-Xaa-Yaa triplet repeats in the triple helical domains of type I collagen chains disrupt helix formation and result in several types of osteogenesis imperfecta*. Human Mutation, 2001. **18**(4): p. 319-326.
19. Pace, J.M., et al., *Disruption of one intra-chain disulphide bond in the carboxyl-terminal propeptide of the proalpha1(I) chain of type I procollagen permits slow assembly and secretion of overmodified, but stable procollagen trimers and results in mild osteogenesis imperfecta*. Journal of Medical Genetics, 2001. **38**(7): p. 443-9.
20. Augusciak-Duma, A., et al., *Mutations in the COL1A1 and COL1A2 genes associated with osteogenesis imperfecta (OI) types I or III*. Acta Biochim Pol, 2018. **65**(1): p. 79-86.
21. Lindert, U., et al., *Insight into the Pathology of a COL1A1 Signal Peptide Heterozygous Mutation Leading to Severe Osteogenesis Imperfecta*. Calcified Tissue International, 2018. **102**(3): p. 373-379.
22. Takeyari, S., et al., *4-Phenylbutyric acid enhances the mineralization of osteogenesis imperfecta iPSC-derived osteoblasts*. Journal of Biological Chemistry, 2021. **296**.

Reference for Qualitative data for melting temperature outcome

1. Wenstrup, R.J., P. Tsipouras, and P.H. Byers, *Osteogenesis imperfecta type IV. Biochemical confirmation of genetic linkage to the pro alpha 2(I) gene of type I collagen*. Journal of Clinical Investigation, 1986. **78**(6): p. 1449-55.
2. Wenstrup, R.J., A.G. Hunter, and P.H. Byers, *Osteogenesis imperfecta type IV: evidence of abnormal triple helical structure of type I collagen*. Human Genetics, 1986. **74**(1): p. 47-53.
3. Willing, M.C., et al., *Heterozygosity for a large deletion in the alpha 2(I) collagen gene has a dramatic effect on type I collagen secretion and produces perinatal lethal osteogenesis imperfecta*. Journal of Biological Chemistry, 1988. **263**(17): p. 8398-404.
4. Byers, P.H., et al., *A novel mutation causes a perinatal lethal form of osteogenesis imperfecta. An insertion in one alpha 1(I) collagen allele (COL1A1)*. Journal of Biological Chemistry, 1988. **263**(16): p. 7855-61.
5. Tenni, R., et al., *Type I procollagen in the severe non-lethal form of osteogenesis imperfecta. Defective pro-alpha 1(I) chains in a patient with abnormal proteoglycan metabolism and mineral deposits in the dermis*. Human Genetics, 1988. **79**(3): p. 245-50.
6. Royce, P.M., et al., *Lethal osteogenesis imperfecta: abnormal collagen metabolism and biochemical characteristics of hypophosphatasia*. European Journal of Pediatrics, 1988. **147**(6): p. 626-31.
7. Bateman, J.F.C., D. Lamande, Mascara, T. Cole, W. G., *Biochemical Heterogeneity of type I collagen mutation in osteogenesis imperfecta*. Annals of the New York Academy of Sciences, 1988.
8. Superti-Furga, A., et al., *Delayed triple-helix formation of abnormal type I collagen is corrected by reduced temperature. Studies of a family with variable expression of osteogenesis imperfecta*. Annals of the New York Academy of Sciences, 1988. **543**: p. 85-92.

9. Wenstrup, R.J., et al., *Arginine for glycine substitution in the triple-helical domain of the products of one alpha2(I) collagen allele (COL1A2) produces the osteogenesis imperfecta type IV phenotype*. Journal of Biological Chemistry, 1988. **263**(16): p. 7734-7740.
10. Pack, M., et al., *Substitution of serine for alpha1(I)-glycine 844 in a severe variant of osteogenesis imperfecta minimally destabilizes the triple helix of type I procollagen. The effects of glycine substitutions on thermal stability are either position or amino acid specific*. Journal of Biological Chemistry, 1989. **264**(33): p. 19694-19699.
11. Starman, B.J., et al., *Osteogenesis imperfecta. The position of substitution for glycine by cysteine in the triple helical domain of the Proalpha1(I) chains of type I collagen determines the clinical phenotype*. Journal of Clinical Investigation, 1989. **84**(4): p. 1206-1214.
12. Wallis, G.A., et al., *Substitution of arginine for glycine at position 847 in the triple-helical domain of the alpha 1 (I) chain of type I collagen produces lethal osteogenesis imperfecta. Molecules that contain one or two abnormal chains differ in stability and secretion*. Journal of Biological Chemistry, 1990. **265**(30): p. 18628-33.
13. Grange, D.K., M.B. Lewis, and J.C. Marini, *Analysis of cultured chorionic villi in a case of osteogenesis imperfecta type II: Implications for prenatal diagnosis*. American Journal of Medical Genetics, 1990. **36**(2): p. 258-264.
14. Westerhausen, A., J. Kishi, and D.J. Prockop, *Mutations that substitute serine for glycine alpha1-598 and glycine alpha1-631 in type I procollagen. The effects of thermal unfolding of the triple helix are position-specific and demonstrate that the protein unfolds through a series of cooperative blocks*. Journal of Biological Chemistry, 1990. **265**(23): p. 13995-14000.
15. Deak, S.B., et al., *The substitution of arginine for glycine 85 of the alpha 1(I) procollagen chain results in mild osteogenesis imperfecta. The mutation provides direct evidence for three discrete domains of cooperative melting of intact type I collagen*. Journal of Biological Chemistry, 1991. **266**(32): p. 21827-32.
16. Steinmann, B., et al., *Substitution of cysteine for glycine-alpha 1-691 in the pro alpha 1(I) chain of type I procollagen in a proband with lethal osteogenesis imperfecta destabilizes the triple helix at a site C-terminal to the substitution*. The Biochemical journal, 1991. **279**: p. 747-52.
17. Tenni, R., et al., *Phenotypic variability and abnormal type I collagen unstable at body temperature in a family with mild dominant osteogenesis imperfecta*. Journal of inherited metabolic disease, 1991. **14**(2): p. 189-201.
18. Tsuneyoshi, T., et al., *Substitutions for glycine alpha 1-637 and glycine alpha 2-694 of type I procollagen in lethal osteogenesis imperfecta. The conformational strain on the triple helix introduced by a glycine substitution can be transmitted along the helix*. The Journal of biological chemistry, 1991. **266**(24): p. 15608-13.
19. Wallis, G.A., et al., *A tripeptide deletion in the triple-helical domain of the pro alpha 1(I) chain of type I procollagen in a patient with lethal osteogenesis imperfecta does not alter cleavage of the molecule by N-proteinase*. Journal of Biological Chemistry, 1992. **267**(35): p. 25529-34.
20. Fertala, A., et al., *Two cysteine substitutions in procollagen I: a glycine replacement near the N-terminus of alpha 1(I) chain causes lethal osteogenesis imperfecta and a glycine replacement in the alpha 2(I) chain markedly destabilizes the triple helix*. Biochemical Journal, 1993. **289**(Pt 1): p. 195-9.
21. Mottes, M., et al., *Paternal mosaicism for a COL1A1 dominant mutation (alpha 1 Ser-415) causes recurrent osteogenesis imperfecta*. Human Mutation, 1993. **2**(3): p. 196-204.

22. Superti-Furga, A., et al., *An intronic deletion leading to skipping of exon 21 of COL1A2 in a boy with mild osteogenesis imperfecta*. Connective Tissue Research, 1993. **29**(1): p. 31-40.
23. Valli, M., et al., *Osteogenesis imperfecta and type-I collagen mutations. A lethal variant caused by a Gly910-->Ala substitution in the alpha 1 (I) chain*. European Journal of Biochemistry, 1993. **211**(3): p. 415-9.
24. Chessler, S.D., G.A. Wallis, and P.H. Byers, *Mutations in the carboxyl-terminal propeptide of the proalpha1(I) chain of type I collagen result in defective chain association and produce lethal osteogenesis imperfecta*. Journal of Biological Chemistry, 1993. **268**(24): p. 18218-18225.
25. Zhuang, J., et al., *Deletion of 19 base pairs in intron 13 of the gene for the proalpha2(I) chain of type-I procollagen (COL1A2) causes exon skipping in a proband with type-I osteogenesis imperfecta*. Human Genetics, 1993. **91**(3): p. 210-216.
26. Bateman, J.F., et al., *A 5' splice site mutation affecting the pre-mRNA splicing of two upstream exons in the collagen COL1A1 gene. Exon 8 skipping and altered definition of exon 7 generates truncated pro alpha 1(I) chains with a non-collagenous insertion destabilizing the triple helix*. The Biochemical journal, 1994. **302**: p. 729-35.
27. Lightfoot, S.J., et al., *Substitution of serine for glycine 883 in the triple helix of the pro alpha 1 (I) chain of type I procollagen produces osteogenesis imperfecta type IV and introduces a structural change in the triple helix that does not alter cleavage of the molecule by procollagen N-proteinase*. The Journal of biological chemistry, 1994. **269**(48): p. 30352-7.
28. Pace, J.M., et al., *Deletions and duplications of Gly-Xaa-Yaa triplet repeats in the triple helical domains of type I collagen chains disrupt helix formation and result in several types of osteogenesis imperfecta*. Human Mutation, 2001. **18**(4): p. 319-326.
29. Pace, J.M., et al., *Disruption of one intra-chain disulphide bond in the carboxyl-terminal propeptide of the proalpha1(I) chain of type I procollagen permits slow assembly and secretion of overmodified, but stable procollagen trimers and results in mild osteogenesis imperfecta*. Journal of Medical Genetics, 2001. **38**(7): p. 443-9.
30. Galika, A.W., Slawomir. Gindzinski, Andrziej, *Studies on type I collagen in skin fibroblasts cultured from twins with lethal osteogenesis imperfecta*. Acta Biochimica Polonica, 2003. **50**(2): p. 481-488.

Reference for Quantitative data for folding outcome

1. Barnes, A.M., et al., *COL1A1 C-propeptide mutations cause ER mislocalization of procollagen and impair C-terminal procollagen processing*. Biochimica et Biophysica Acta (BBA) - Molecular Basis of Disease, 2019. **1865**(9): p. 2210-2223.

Reference for Quantitative data for secretion outcome

1. Barsh, G.S., K.E. David, and P.H. Byers, *Type I osteogenesis imperfecta: a nonfunctional allele for pro alpha 1 (I) chains of type I procollagen*. Proceedings of the National Academy of Sciences of the United States of America, 1982. **79**(12): p. 3838-42.
2. Steinmann, B., et al., *Cysteine in the triple-helical domain of one allelic product of the alpha 1(I) gene of type I collagen produces a lethal form of osteogenesis imperfecta*. Journal of Biological Chemistry, 1984. **259**(17): p. 11129-38.

3. Bonaventure, J., et al., *Abnormal procollagen synthesis in fibroblasts from three patients of the same family with a severe form of osteogenesis imperfecta (type III)*. Biochimica et Biophysica Acta, 1986. **889**(1): p. 23-34.
4. Wenstrup, R.J., P. Tsipouras, and P.H. Byers, *Osteogenesis imperfecta type IV. Biochemical confirmation of genetic linkage to the pro alpha 2(I) gene of type I collagen*. Journal of Clinical Investigation, 1986. **78**(6): p. 1449-55.
5. Royce, P.M., et al., *Lethal osteogenesis imperfecta: abnormal collagen metabolism and biochemical characteristics of hypophosphatasia*. European Journal of Pediatrics, 1988. **147**(6): p. 626-31.
6. Forlino, A., et al., *Severe (type III) osteogenesis imperfecta due to glycine substitutions in the central domain of the collagen triple helix*. Human Molecular Genetics, 1994. **3**(12): p. 2201-2206.
7. Besio, R., et al., *4-PBA ameliorates cellular homeostasis in fibroblasts from osteogenesis imperfecta patients by enhancing autophagy and stimulating protein secretion*. Biochimica et Biophysica Acta (BBA) - Molecular Basis of Disease, 2018. **1864**(5, Part A): p. 1642-1652.

Reference for Meta-Analysis

1. Kirsch, E., et al., *Disorder of collagen metabolism in a patient with osteogenesis imperfecta (lethal type): increased degree of hydroxylation of lysine in collagen types I and III*. European Journal of Clinical Investigation, 1981. **11**(1): p. 39-47.
2. Steinmann, B., et al., *Cysteine in the triple-helical domain of one allelic product of the alpha 1(I) gene of type I collagen produces a lethal form of osteogenesis imperfecta*. Journal of Biological Chemistry, 1984. **259**(17): p. 11129-38.
3. de Vries, W.N. and W.J. de Wet, *The molecular defect in an autosomal dominant form of osteogenesis imperfecta. Synthesis of type I procollagen containing cysteine in the triple-helical domain of pro-alpha 1(I) chains*. Journal of Biological Chemistry, 1986. **261**(19): p. 9056-64.
4. Vogel, B.E., et al., *A point mutation in a type I procollagen gene converts glycine 748 of the alpha 1 chain to cysteine and destabilizes the triple helix in a lethal variant of osteogenesis imperfecta*. Journal of Biological Chemistry, 1987. **262**(30): p. 14737-44.
5. Constantinou, C.D., et al., *The A and B fragments of normal type I procollagen have a similar thermal stability to proteinase digestion but are selectively destabilized by structural mutations*. European journal of biochemistry, 1987. **163**(2): p. 247-51.
6. Tenni, R., et al., *Type I procollagen in the severe non-lethal form of osteogenesis imperfecta. Defective pro-alpha 1(I) chains in a patient with abnormal proteoglycan metabolism and mineral deposits in the dermis*. Human Genetics, 1988. **79**(3): p. 245-50.
7. Rao, V.H., et al., *Decreased thermal denaturation temperature of osteogenesis imperfecta mutant collagen is independent of post-translational overmodifications of lysine and hydroxylysine*. Journal of Biological Chemistry, 1989. **264**(3): p. 1793-8.
8. Baker, A.T., et al., *Changes in collagen stability and folding in lethal perinatal osteogenesis imperfecta. The effect of α1(I)-chain glycine-to-arginine substitutions*. Biochemical Journal, 1989. **261**(1): p. 253-257.
9. Constantinos D. Constantinou, K.B.N., * and D.J. Prockop, *A lethal variant of osteogenesis imperfecta has a single base mutation that substitutes cysteine for glycine 904 of the alpha 1(I) chain of type I procollagen. The asymptomatic mother has an unidentified mutation producing an overmodified and unstable type I procollagen*. J Clin Invest, 1989.

10. Baldwin, C.T., et al., *A single base mutation that converts glycine 907 of the alpha 2(I) chain of type I procollagen to aspartate in a lethal variant of osteogenesis imperfecta. The single amino acid substitution near the carboxyl terminus destabilizes the whole triple helix*. The Journal of biological chemistry, 1989. **264**(5): p. 3002-6.
11. Valli, M., R. Tenni, and G. Cetta, *Moderately Severe Osteogenesis Imperfecta: Biochemical Studies Showing Variable Defect Localization in the Triple-Helical Domain of Type I Collagen*. Matrix, 1990. **10**(3): p. 200-205.
12. Valli, M., et al., *A de novo G to T transversion in a pro-alpha 1 (I) collagen gene for a moderate case of osteogenesis imperfecta. Substitution of cysteine for glycine 178 in the triple helical domain*. Journal of Biological Chemistry, 1991. **266**(3): p. 1872-8.
13. Bateman, J.F., et al., *Characterization of a type I collagen alpha 2(I) glycine-586 to valine substitution in osteogenesis imperfecta type IV. Detection of the mutation and prenatal diagnosis by a chemical cleavage method*. Biochemical Journal, 1991. **276**(Pt 3): p. 765-70.
14. Wenstrup, R.J., et al., *The effects of different cysteine for glycine substitutions within alpha 2(I) chains. Evidence of distinct structural domains within the type I collagen triple helix*. The Journal of biological chemistry, 1991. **266**(4): p. 2590-4.
15. Bonaventure, J., et al., *A dominant mutation in the COL1A1 gene that substitutes glycine for valine causes recurrent lethal osteogenesis imperfecta*. Human Genetics, 1992. **89**(6): p. 640-6.
16. Bateman, J.F., et al., *Lethal perinatal osteogenesis imperfecta due to a type I collagen alpha 2(I) Gly to Arg substitution detected by chemical cleavage of an mRNA:cDNA sequence mismatch*. Human Mutation, 1992. **1**(1): p. 55-62.
17. Bateman, J.F., et al., *Characterization of three osteogenesis imperfecta collagen alpha 1(I) glycine to serine mutations demonstrating a position-dependent gradient of phenotypic severity*. The Biochemical journal, 1992. **288**: p. 131-5.
18. Edwards, M.J., et al., *Recurrence of lethal osteogenesis imperfecta due to parental mosaicism for a mutation in the COL1A2 gene of type I collagen. The mosaic parent exhibits phenotypic features of a mild form of the disease*. Human mutation, 1992. **1**(1): p. 47-54.
19. Mottes, M., et al., *Paternal mosaicism for a COL1A1 dominant mutation (alpha 1 Ser-415) causes recurrent osteogenesis imperfecta*. Human Mutation, 1993. **2**(3): p. 196-204.
20. Brenner, R.E., et al., *Defective collagen fibril formation and mineralization in osteogenesis imperfecta with congenital joint contractures (Bruck syndrome)*. European Journal of Pediatrics, 1993. **152**(6): p. 505-8.
21. Valli, M., et al., *Osteogenesis imperfecta and type-I collagen mutations. A lethal variant caused by a Gly910-->Ala substitution in the alpha 1 (I) chain*. European Journal of Biochemistry, 1993. **211**(3): p. 415-9.
22. Vetter, U., et al., *Collagen crosslinks and mineral crystallinity in bone of patients with osteogenesis imperfecta*. Journal of Bone and Mineral Research, 1993. **8**(2): p. 133-137.
23. Kurosaka, D., et al., *Substitution of cysteine for glycine-946 in the alpha1(I) chain of type I procollagen causes lethal osteogenesis imperfecta*. Journal of Biochemistry, 1994. **115**(5): p. 853-857.
24. Raghunath, M., P. Bruckner, and B. Steinmann, *Delayed triple helix formation of mutant collagen from patients with osteogenesis imperfecta*. Journal of molecular biology, 1994. **236**(3): p. 940-9.
25. Sarafova, A.P., et al., *Three novel type I collagen mutations in osteogenesis imperfecta type IV probands are associated with discrepancies between electrophoretic migration of osteoblast and fibroblast collagen*. Human mutation, 1998. **11**(5): p. 395-403.

26. Bank, R.A., et al., *Pyridinium cross-links in bone of patients with osteogenesis imperfecta: evidence of a normal intrafibrillar collagen packing.* Journal of Bone & Mineral Research, 2000. **15**(7): p. 1330-6.
27. Cabral, W.A., E.J. Chernoff, and J.C. Marini, *G76E substitution in type I collagen is the first nonlethal glutamic acid substitution in the alpha1(I) chain and alters folding of the N-terminal end of the helix.* Molecular genetics and metabolism, 2001. **72**(4): p. 326-35.
28. Cabral, W.A., et al., *Type I collagen triplet duplication mutation in lethal osteogenesis imperfecta shifts register of alpha chains throughout the helix and disrupts incorporation of mutant helices into fibrils and extracellular matrix.* The Journal of biological chemistry, 2003. **278**(12): p. 10006-12.
29. Cabral, W.A., et al., *Y-position cysteine substitution in type I collagen (alpha1(I) R888C/p.R1066C) is associated with osteogenesis imperfecta/Ehlers-Danlos syndrome phenotype.* Human mutation, 2007. **28**(4): p. 396-405.
30. Kruczek, A., et al., *Two novel COL1A1 mutations in patients with osteogenesis imperfecta (OI) affect the stability of the collagen type I triple-helix.* Journal of Applied Genetics, 2008. **49**(3): p. 283-295.
31. Makareeva, E., et al., *Structural heterogeneity of type I collagen triple helix and its role in osteogenesis imperfecta.* Journal of Biological Chemistry, 2008. **283**(8): p. 4787-4798.
32. Taga, Y., et al., *Site-specific Quantitative Analysis of Overglycosylation of Collagen in Osteogenesis Imperfecta Using Hydrazide Chemistry and SILAC.* Journal of Proteome Research, 2013. **12**(5): p. 2225-2232.
33. Mirigian, L.S., E. Makareeva, and S. Leikin, *Pulse-chase analysis of procollagen biosynthesis by azidohomoalanine labeling.* Connective Tissue Research, 2014. **55**(5-6): p. 403-10.
34. Makareeva, E., et al., *Substitutions for arginine at position 780 in triple helical domain of the alpha1(I) chain alter folding of the type I procollagen molecule and cause osteogenesis imperfecta.* PLoS ONE, 2018. **13**(7): p. e0200264.
35. Sarathchandra, P., F.M. Pope, and S.Y. Ali, *Morphometric Analysis of Type I Collagen Fibrils in the Osteoid of Osteogenesis Imperfecta.* Calcified Tissue International, 1999. **65**(5): p. 390-395.
36. Vomund, A.N., et al., *Potential modifier role of the R618Q variant of proα2(I)collagen in type I collagen fibrillogenesis: in vitro assembly analysis.* Molecular Genetics and Metabolism, 2004. **82**(2): p. 144-153.
37. Balasubramanian, M., et al., *Ultrastructural and histological findings on examination of skin in osteogenesis imperfecta: a novel study.* Clin Dysmorphol, 2015. **24**(2): p. 45-54.
38. Jones, C.J.P., et al., *Collagen Defect of Bone in Osteogenesis Imperfecta (Type I): An Electron Microscopic Study.* Clinical Orthopaedics and Related Research®, 1984. **183**.
39. Stöss, H. and P. Freisinger, *Collagen fibrils of osteoid in osteogenesis imperfecta: Morphometrical analysis of the fibril diameter.* American Journal of Medical Genetics, 1993. **45**(2): p. 257-257.
40. Barnes, A.M., et al., *COL1A1 C-propeptide mutations cause ER mislocalization of procollagen and impair C-terminal procollagen processing.* Biochimica et Biophysica Acta (BBA) - Molecular Basis of Disease, 2019. **1865**(9): p. 2210-2223.
41. Cassella, J.P. and S.Y. Ali, *Abnormal collagen and mineral formation in osteogenesis imperfecta.* Bone and Mineral, 1992. **17**(2): p. 123-128.
42. Cassella, J.P., et al., *A Morphometric analysis of osteoid collagen fibril diameter in osteogenesis imperfecta.* Bone, 1994. **15**(3): p. 329-334.
43. Lindahl, K., et al., *COL1 C-propeptide cleavage site mutations cause high bone mass osteogenesis imperfecta.* Human Mutation, 2011. **32**(6): p. 598-609.
